# A window into lysogeny: revealing temperate phage biology with transcriptomics

**DOI:** 10.1099/mgen.0.000330

**Published:** 2020-02-05

**Authors:** Siân V. Owen, Rocío Canals, Nicolas Wenner, Disa L. Hammarlöf, Carsten Kröger, Jay C. D. Hinton

**Affiliations:** ^1^​ Department of Biomedical Informatics, Harvard Medical School, Boston, MA, USA; ^2^​ Institute of Integrative Biology, University of Liverpool, Liverpool, UK; ^3^​ Science for Life Laboratory, KTH, Stockholm, Sweden; ^4^​ Department of Microbiology, School of Genetics and Microbiology, Moyne Institute of Preventive Medicine, Trinity College Dublin, Dublin 2, Ireland; ^†^​ Present address: GSK Vaccines Institute for Global Health, Siena, Italy

**Keywords:** bacteriophage, transcriptomics, RNA-seq, lysogeny, prophage

## Abstract

Prophages are integrated phage elements that are a pervasive feature of bacterial genomes. The fitness of bacteria is enhanced by prophages that confer beneficial functions such as virulence, stress tolerance or phage resistance, and these functions are encoded by ‘accessory’ or ‘moron’ loci. Whilst the majority of phage-encoded genes are repressed during lysogeny, accessory loci are often highly expressed. However, it is challenging to identify novel prophage accessory loci from DNA sequence data alone. Here, we use bacterial RNA-seq data to examine the transcriptional landscapes of five *Salmonella* prophages. We show that transcriptomic data can be used to heuristically enrich for prophage features that are highly expressed within bacterial cells and represent functionally important accessory loci. Using this approach, we identify a novel antisense RNA species in prophage BTP1, STnc6030, which mediates superinfection exclusion of phage BTP1. Bacterial transcriptomic datasets are a powerful tool to explore the molecular biology of temperate phages.

## Data Summary

All accession numbers for the data used in this study can be found in Table S1 (available with the online version of this article).

**Impact Statement**

Here, we demonstrate that bacterial transcriptomic data are a powerful tool for discovering novel temperate phage biology, specifically for identifying prophage accessory and regulatory loci. Despite the fact that much of the knowledge underpinning modern genetic and genomic technologies originates from the study of phage, 'omic' technologies have ironically not yet been widely adopted in the phage biology field. Our transcriptomic approach is broadly applicable to prophages in all bacteria and existing publicly available transcriptomic data. We anticipate that adoption of this approach could bring new insights into the fundamental biology of temperate phages, particularly in terms of host modulation and regulatory architecture.

## Introduction

Temperate bacteriophages can integrate into the genome of host bacteria (lysogeny), and persist as vertically inherited genomic elements known as prophages. The majority of the temperate phage genome encodes proteins dedicated to virion production and host cell lysis, functions that are toxic to bacterial cells. To exist stably as passive genomic elements, the expression of the majority of prophage genes must be repressed at the transcriptional level. Only those functions necessary to maintain and favour lysogeny are expressed. The molecular mechanisms governing prophage gene regulation are best understood in the archetypical *Escherichia coli* phage lambda (λ), in which the synthesis of a single protein, the CI repressor, is sufficient to maintain the lysogenic state of the prophage [1]. Molecular studies investigating the gene expression of phage lambda lysogens showed that four additional lambda genes are expressed from the integrated prophage genome during lysogenic growth: the *rexAB* operon, encoding a superinfection immunity system; and the *lom* and *bor* genes, which encode virulence factors [2–6].

The expression of fitness-associated prophage-encoded genes during lysogeny represents a mutualism whereby enhancing the fitness of the bacterial host directly increases the fitness (as measured by genome replication) of the integrated prophage [7]. Such genes, known as 'accessory' or 'moron' loci [8], with diverse functions such as virulence, stress resistance and phage resistance, have been found in numerous characterized bacterial prophages [7, 9, 10]. Importantly, prophage accessory loci are not required for any part of the phage life cycle, and only affect the biology of the host bacterium during lysogeny, a process known as lysogenic conversion. In *Salmonella enterica* subsp. *enterica* serovar Typhimurium, and many other bacterial pathogens, prophage-encoded accessory loci are important for virulence in animal models [11–14].

The majority of bacterial taxa contain prophages [15, 16], and prophages are very frequently the main sources of genetic diversity between closely related bacterial strains or pathovariants [17, 18]. Given that more genome sequences exist for bacteria than for any other domain of life, and bacteria frequently harbour multiple prophages, it has been argued that temperate phage may be the most deeply sequenced organisms on the planet [19]. Prophages, therefore, represent a vast and largely unexplored reservoir of functionally important genes. However, the identification of prophage accessory loci in bacterial genome sequences is challenging, particularly in bacterial taxa in which prophages have not been well characterized. Prophage-encoded genes are generally annotated as hypothetical proteins; based on DNA sequence data alone, it is difficult to distinguish the hypothetical proteins that represent novel accessory genes from those that represent divergent genes involved in the phage lifecycle.

Here, we use transcriptome-based gene expression profiling to show that prophage accessory loci typically have unique transcriptional signatures that contrast to phage lytic genes, an observation which can be exploited to heuristically enrich for novel prophage accessory features and genes involved in the maintenance of lysogeny. Our transcriptomic data revealed an uncharacterized antisense RNA (asRNA) of *S *. *enterica* serovar Typhimurium prophage BTP1 that functions as a novel superinfection-exclusion factor. In summary, the gene expression profiles of prophage regions are a powerful tool to discover novel temperate phage biology simply by identifying the subset of prophage genetic features that are highly expressed in lysogenic cells.

## Methods

### Transcriptomic analysis of the prophage regions of *S *. *enterica* serovar Typhimurium D23580

Prophage gene expression during lysogeny was investigated using data from three previous studies [20–22]. A description of the experimental conditions associated with the RNA-seq data used in this analysis is given in Table S2. Transcripts per million (TPM) values were obtained from Canals *et al*. and represent a normalized expression value per gene and condition [20, 23, 24]. As previously described, a TPM cut-off score of 10 was used to define gene expression; only genes with a TPM value of >10 were considered to be expressed [22]. Heat maps showing absolute expression were obtained using TPM values and conditional formatting in Microsoft Excel. Prophage transcriptome maps were generated by visualization of sequence reads using the Integrated Genome Browser (igb) [25]. For display in igb, the read depth was adjusted in relation to the cDNA library with the lowest number of reads [26]. For identification of prophage regulatory or accessory genes, a TPM cut-off score of 100 was used to define high expression and only prophage genes with a TPM value of >100 were considered to be highly expressed and, therefore, putatively involved in prophage lysogeny regulation or accessory functions.

### Plasmid construction

All oligonucleotide (primer) sequences used in this study are listed in Table S3, and bacterial strains, phages and plasmids are given in Table S4. The plasmid to overexpress the STnc6030 asRNA (pP_L_-STnc6030) was constructed using the overlap-extension PCR cloning method [27].

The pJV300 (pP_L_) plasmid [28] was initially modified to encode gentamicin resistance by overlap-extension PCR cloning, and the resulting plasmid was named pP_L_-Gm (Table S4). Chimeric primers NW_88 and NW_89, containing pJV300 plasmid sequence at the 5′ end and insert sequence at the 3′ end, were first used to PCR amplify the *aacC1* gentamicin-resistance locus from the pME4510 plasmid [29]. A 30 ng sample of the template plasmid was mixed with 150–300 ng insert, 10 µlQ5 buffer, 1 µl dNTPs, 0.5 µl Q5 DNA polymerase (New England Biolabs), and water was added to a final volume of 50 µl. PCR reactions were carried out as follows: 98 °C, 30 s; 25× (98 °C, 10 s; 55 °C, 30 s, 72 °C, 3 min); 72 °C, 5 min. The original plasmid template was then digested using the restriction enzyme *Dpn*I in Cutsmart buffer (1×) (New England Biolabs), according to the manufacturer’s instructions, and the overlap-extension PCR products were used to transform chemically competent *E. coli* TOP10 cells [30]. Cells harbouring the new pP_L_-Gm (pJV300 gentamicin-resistant derivative) plasmid were selected by plating on LB agar containing gentamicin (20 µg ml^−1^). Overlap-extension PCR cloning was subsequently used to insert a sequence of interest into the pP_L_-Gm plasmid downstream of the P_LlacO-1_ constitutive promoter. The same procedure previously described was followed, except that the chimeric primers used to amplify the insert were NW_295 and NW_296, and D23580 genomic DNA was used as a template. These primers targeted the region of the pP_L_-Gm plasmid between the P_LlacO-1_ promoter and the *rrnB* transcriptional terminator. To confirm that the plasmid carried the correct construction after transformation, primers external to the insertion site were used to Sanger sequence the inserted fragment (GATC Biotech).

### GFP reporter strain construction

To generate a transcriptional gene fusion to serve as a marker of prophage induction, a promoter-less *gfp^+^* gene was used to replace the lysis genes of prophage BTP1. In order to insert the *gfp^+^* gene into the chromosome, a construct was designed based on the *gfp^+^* gene of plasmid pZEP08 (Hautefort et al., 2003), and the FRT-site-flanked kanamycin gene of the pKD4 plasmid. Primers were designed so that the orientation of the kanamycin gene was opposite to that of the *gfp^+^*, to minimize the likelihood of polar effects from transcription of the kanamycin resistance gene. Primers Late_gfp_2_L_f and gfp_kan_2_L_r were used to amplify the *gfp^+^* gene using pZEP08 DNA as the template, and primers Late_gfp_2_R_r and gfp_kan_2_R_f were used to amplify the kanamycin resistance locus. A third overlap-extension PCR reaction was used to fuse both DNA fragments together. The construct was extracted from agarose gel and electroporated into D23580 (pSIM5-tet) (Table S4) electrocompetent cells, and recombinants were selected on LB agar containing kanamycin (50 µg ml^−1^) as previously described [31].

### Microscopy

An EVOS FL cell imaging system (Thermo Fisher), fitted with a GFP light cube (470/22 nm excitation, 525/50 nm emission), was used to visualize cells under transmitted light and fluorescent light with an Olympus 100× super-apochromat, coverslip-corrected oil objective (Thermo Fisher; AMEP4733). Cell samples (2 µl) were pipetted onto glass microscopy slides and covered with a glass coverslip.

### Extraction of total bacterial RNA

Bacterial RNA was extracted as previously described [22, 32]. Four or five OD_600_ units were removed from bacterial cultures, and cellular transcription was stopped using 0.4× culture volume of a 5 % phenol (pH 4.3), 95 % ethanol ‘stop’ solution (Sigma Aldrich; P4557 and E7023, respectively). Cells were stabilized on ice in stop solution for at least 30 min before being harvested at 7000 ***g*** for 10 min at 4 °C. At this point, pellets were either stored at −80 °C, or RNA was immediately extracted.

To isolate RNA, pellets were resuspended in 1 ml TRIzol reagent (Invitrogen). Chloroform (400 μl) was added and the samples were immediately and thoroughly mixed by inversion. Samples were moved to a Phase-lock tube (5 Prime), and the aqueous and organic phases were separated by centrifugation at 16 000 ***g*** for 15 min at room temperature in a bench-top centrifuge. The aqueous phase was transferred into a new 1.5 ml tube and the RNA was precipitated using isopropanol for 30 min at room temperature, followed by centrifugation at 21 000 ***g*** for 30 min at room temperature. The RNA pellet was rinsed with 70 % ethanol, followed by centrifugation at 21 000 ***g*** for 10 min at room temperature. The RNA pellet was air-dried for 15 min and resuspended in DEPC-treated water at 65 °C with shaking at 900 r.p.m. on a Thriller thermoshaker (Peqlab) for 5 min with occasional vortexing. RNA was kept on ice whenever possible and was stored at −80 °C. RNA concentration was measured using a Nanodrop ND-1000 spectrophotometer, and RNA quality was verified visually using a 2100 Bioanalyser (Agilent).

### Detection of STnc6030 by Northern blot

Following extraction, total RNA was separated based on size by electrophoresis through a denaturing 20 mM guanidine thiocyanate, 1.5 % agarose gel in TBE (1×). Generally, 1–10 µg RNA was mixed with an equal volume of 2× urea-blue denaturing buffer (0.025 % xylene cyanol, 0.025 % bromophenol blue and 50 % urea) and samples were heat denatured at 90 °C for 5 min and chilled on ice before loading. Low-range ssRNA ladder (4 µl) (New England Biolabs) or 5 µl RNA molecular weight marker I DIG-labelled (Roche) were treated in the same way as the RNA samples to allow the detected transcript length to be estimated. Samples were run in 1× TBE buffer at a constant voltage of 80 V (at 4 °C).

Separated RNA was transferred from polyacrylamide gels to positively charged nylon membranes (Roche) using a Trans-Blot SD semi-dry electrophoretic transfer cell (BioRad) at a constant amplitude of 125 mA for 30 min at 4 °C. For denaturing agarose gels, separated RNA was transferred to positively charged nylon membranes using overnight capillary transfer in 20× saline-sodium citrate (SSC) buffer, as described in the DIG Northern starter kit manual (Roche).

RNA was UV-crosslinked to the membranes in a CL-1000 UV-crosslinker (UVP) set to 3600 (360 000 μJ cm^−2^). The membrane was equilibrated in hybridization buffer for 1 h at 68 °C in pre-warmed DIG Easy Hyb solution (Roche) in a rotating hybridization oven. Five microlitres (approximately 1.25 µg) riboprobe was heat denatured in 5 ml DIG Easy Hyb solution at 68 °C for 30 min and added to the membrane for hybridization overnight at 68 °C in the rotating hybridization oven. The oligonucleotide sequences used to generate the DIG-labelled riboprobe are given in Table S3. Membrane washing, blocking and transcript detection steps were carried out as described in the Roche manual. An ImageQuant LAS4000 Imager was used for blot detection.

### Phage enumeration and plaquing

Phage enumeration and plaque isolations were carried out using the double layer agar technique. To count spontaneously induced phages in overnight cultures, 1 ml overnight culture was passed through a 0.22 µm syringe filter. For enumeration, for phage lysates or culture supernatants, serial 10-fold dilutions were made in sterile lysogeny broth (LB) (Lennox formulation: 10 g tryptone l^−1^, 5 g yeast extract l^−1^, 5 g NaCl l^−1^). For overnight culture supernatant, dilution up to 10^−7^ was sufficient, whereas for phage lysates, higher dilutions were required (typically up to 10^−10^). Four millilitres 0.4 % LB agar were seeded with 100 µl overnight culture of the required indicator strain (approximately 10^8^ c.f.u.) and, once solidified, phage dilutions were applied to the agar in 10 µl drops. After drying for 30 mins, plates were incubated overnight at 37 °C. Phage concentrations were calculated as plaque forming units per ml (p.f.u. ml^−1^) of lysate or culture supernatant.

### Isolation and sequencing of STnc6030 escape phage

One hundred microlitres of 10-fold dilutions of high titre BTP1 stock (10^11^ p.f.u. ml^−1^) were mixed with 100 µl D23580 ΔBTP1 pP_L_-STnc6030, added to 3 ml molten LB (Lennox) 0.3 % agar and poured onto LB plates. The plates were incubated right-side up overnight at 37 °C for 16 h. The frequency of occurrence of spontaneous STnc6030 ‘escape’ mutants was determined as the p.f.u. ml^−1^ of escape mutants divided by the input titre of BTP1 phage. Escape phage plaques were picked and replicated on D23580 ΔBTP1 pP_L_-STnc6030. To ensure purity, a nested PCR strategy was used to ensure amplification of the STnc6030 region from the escape phages without amplification of WT STnc6030 from contaminating pP_L_-STnc6030 plasmid DNA. The larger STnc6030 region was first amplified using oligonucleotides NW_296 and NW_298, and this amplicon was used for a more targeted amplification of the STnc6030 region using oligonucleotides NW_296 and NW_295, yielding a 787 bp amplicon that was Sanger sequenced (GATC Biotech). All amplicons were sequenced with oligonucleotides NW_296 and NW_295, so that the entire length of the STnc6030 region could be resolved. SNPs were detected by alignment of the Sanger sequencing data with the STnc6030 sequence from WT *S *. *enterica* serovar Typhimurium D23580 chromosome (GenBank accession no. FN424405) using SnapGene software (from GSL Biotech; available at www.snapgene.com).

## Results

The *S *. *enterica* serovar Typhimurium strain D23580 is a representative of the sequence type (ST) 313 lineage 2, which is currently responsible for the epidemic of invasive non-typhoidal salmonellosis in Africa [33]. RNA-seq data from two previous studies [20, 21] are available via the SalComD23580 online resource (https://tinyurl.com/SalComD23580), and were used to interrogate the transcriptomes of the five prophages of *S *. *enterica* serovar Typhimurium D23580 [31]. Additional RNA-seq data for the *S *. *enterica* serovar Typhimurium ST19 strain 4/74 from Kröger *et al*. allowed investigation of the conservation of prophage transcriptional landscapes in independent strain backgrounds [22, 34]. Prophages Gifsy-2, ST64B and Gifsy-1 are present in both the 4/74 and D23580 strains, while BTP1 and BTP5 are exclusive to strain D23580.

Our published RNA-seq experiments generated transcriptomes of bacterial cell populations from the intra-macrophage environment and from growth in 16 different *in vitro* conditions (Table S2) designed to mimic the environments that *Salmonella* experiences during infection of a mammalian host [20, 22, 34]. A differential RNA-seq (dRNA-seq) approach was used to assign transcriptional start sites (TSSs) [35] for a subset of the *in vitro* conditions and the intra-macrophage environment, and these were included in the analysis of the prophage transcriptomes. The identification of TSSs is particularly important for prophage regions to show the position and activity of promoters that control the regulatory architecture of the prophages.

We previously showed that, of the five prophages in *S *. *enterica* serovar Typhimurium D23580, only prophage BTP1 produced infectious viruses [31]. Prophages Gifsy-2, ST64B and Gifsy-1 all contain mutations that either prevent prophage induction (Gifsy-1), or preclude assembly of infectious viruses (ST64B and Gifsy-2). We have never detected infectious BTP5 viruses; therefore, it is not known if the BTP5 prophage is functional. We began by using the RNA-seq data to reveal the transcriptional landscapes of these five distinct *Salmonella* prophages during lysogeny.

### Prophage transcriptional landscapes

#### Prophage BTP1

**Fig. 1.**
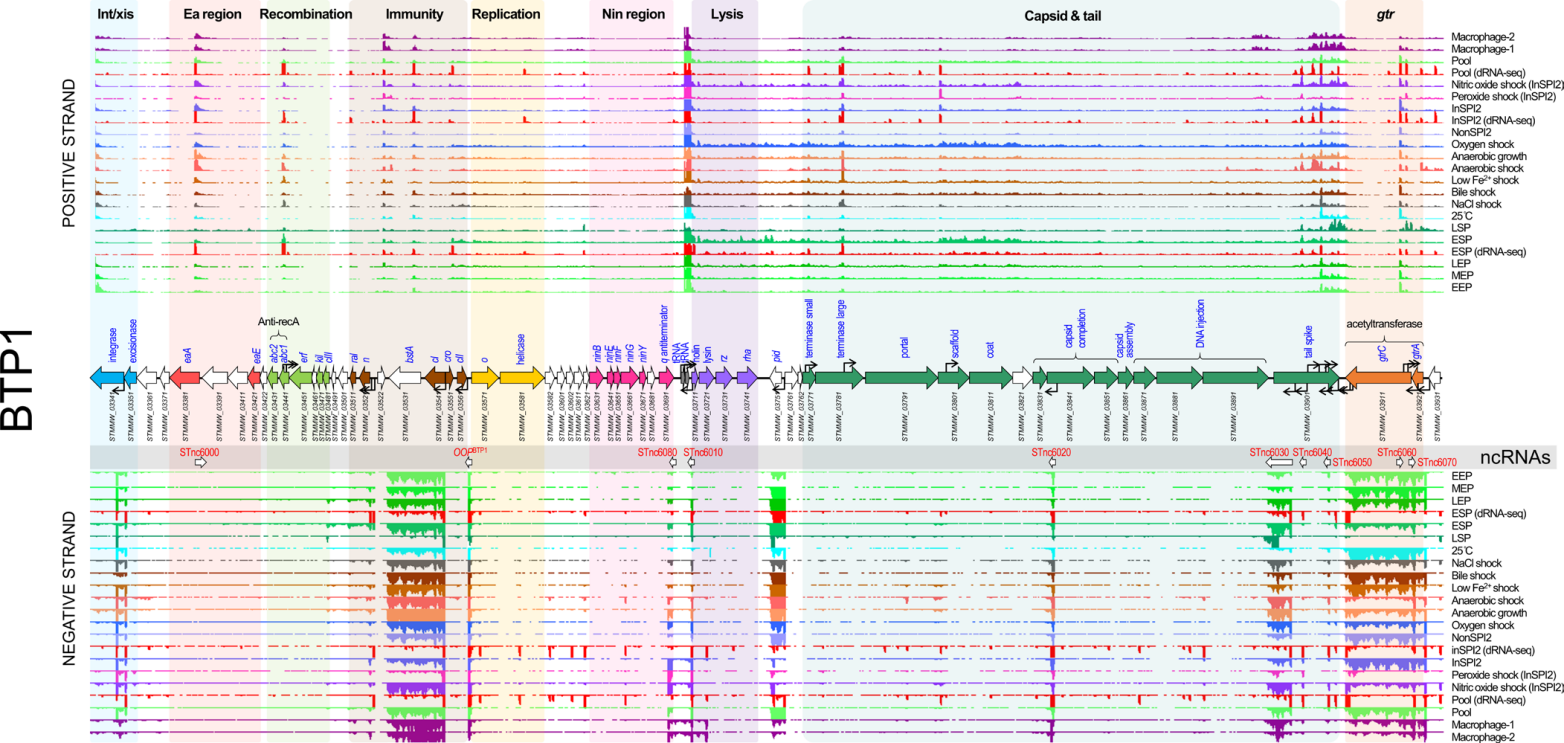
The transcriptomic landscape of the BTP1 prophage of *S *. *enterica* serovar Typhimurium D23580 across 22 different RNA-seq experiments. RNA-seq and dRNA-seq data are from Canals *et al.* [20] and Hammarlöf *et al.* [21]. Each coloured horizontal track represents a different RNA-seq condition (Table S2). The upper panel shows sequence reads mapped to the positive strand and the lower panel reads mapped to the negative strand. The dRNA-seq data are shown in red, and were used to identify the TSSs, which are indicated by curved black arrows on the main annotation track. Annotated phage genes are grouped into functional clusters. Non-coding RNAs are represented beneath the main annotation track.

The transcriptome map (Figs 1 and S1) of the BTP1 lysogen (D23580) showed relatively little transcription, interspersed with a small number of highly transcribed regions. The gene expression data for the BTP1 prophage can be visualized interactively via the SalComD23580 online resource at https://tinyurl.com/SalComD23580-BTP1 and https://tinyurl.com/JBrowseD23580-BTP1. To quantitatively assess prophage gene expression, we used previously established TPM expression values [20]. Genes with TPM values ≤10 were considered not to be expressed. Five genes (excluding the two tRNA genes located in the centre of the prophage) showed particularly high expression (TPM>100; Table S5): *bstA* (*STMMW_03531*, formerly known as *ST313-td*), *cI^BTP1^* (*STMMW_03541*), *pid* (*STMMW_03751*), *gtrC^BTP1^* (*STMMW_03911*) and *gtrA^BTP1^* (*STMMW_03921*). The *cI^BTP1^* and the *bstA* genes represent an operon transcribed from a single TSS, and RNA-seq reads mapped across the 88 bp intergenic region. The *cI^BTP1^-bstA* operon was highly expressed in most conditions, consistent with the CI^BTP1^ protein functioning as the prophage repressor. The *pid* gene, linked to maintenance of the pseudolysogenic state in phage P22 [36, 37] also showed a high level of transcription in the majority of conditions. Lastly, a two-gene operon encoding *gtrA^BTP1^* and *gtrC^BTP1^* showed high expression (TPM>100; Fig. S1). The *gtrA^BTP1^* gene encodes a putative bactoprenol-linked glucosyltranslocase (also known as ‘flippase’), and *gtrC^BTP1^* encodes a putative acetyltransferase that mediates the addition of an acetyl group to the rhamnose subunit of the O-antigen [38]. The *gtr* operon is commonly found in P22-like phages and modifies the O-antigen component of the lipopolysaccharide to inhibit superinfection of the lysogen [39].

The structural (capsid and tail) and lysis genes (late lytic genes) showed low expression levels (TPM 10–20) in the majority of conditions tested. We previously showed that the BTP1 prophage exhibits an extremely high level of spontaneous induction that occurs in 0.2 % of the cellular population [31]. Because the transcriptome data generated by RNA-seq represents the mean gene expression across an entire bacterial population, we propose that the apparent low-level expression of lytic genes (which are involved in cell lysis or components of the capsid and tail) reflects extremely high-level transcription of lytic genes within the fraction of the population in which the BTP1 prophage is spontaneously induced. We confirmed this hypothesis by construction of a lytic gene reporter strain by replacing the BTP1 lysis genes with GFP (D23580 Δlysis::*gfp^+^*). Fluorescence microscopy of this strain revealed a high GFP signal in a small fraction of cells from an overnight culture (indicating that spontaneous BTP1 prophage induction had occurred), whilst no GFP signal was detected in the majority of the cells (Fig. S2).

The BTP1 prophage lytic genes were transcribed in a large polycistronic operon that begins upstream of *STMMW_03711*, directly after the two central tRNA genes. Operons may possess more than one promoter, producing transcripts of different lengths [32]. The BTP1 prophage capsid gene cluster contained four promoters on the coding strand. The BTP1 tail spike gene showed a different transcriptional pattern to the rest of the lytic gene operon driven by several secondary promoters on both the coding and non-coding strand (Fig. 1). A clearer representation of the multiple TSSs associated with the tail spike gene is available at https://tinyurl.com/BTP1-tailspike-TSS.

Nine candidate non-coding RNAs (ncRNAs) were annotated in the transcriptome of BTP1 (Fig. 1). One of these, designated *OOP^BTP1^*, occupies the same position as the *OOP* ncRNA of phage lambda, which is antisense to and overlapping the 3′ end of the *cII* gene [40]. In phage lambda, *OOP* is thought to regulate the expression of the *cII* gene, modulating the switch between lysogenic and lytic infection [41]. Few phages and prophages have been studied at the transcriptomic level; therefore, it is unsurprising that the remaining eight putative ncRNAs have not been previously detected in other lambdoid phages and prophages, and their biological functions remain unknown. One of the identified ncRNAs, STnc6030, was notable due to its unusually large size (786 nt), its position antisense to phage structural genes and its high level of expression (mean TPM=90), and was investigated further (see below).

#### Prophage Gifsy-2

**Fig. 2.**
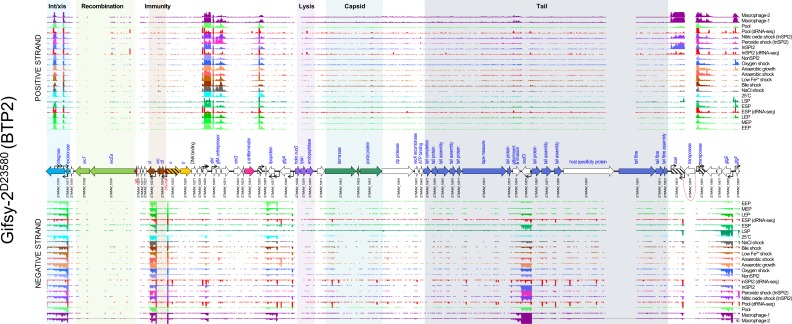
The transcriptomic landscape of the Gifsy-2 prophage of *S *. *enterica* serovar Typhimurium D23580 across 22 different RNA-seq experiments. RNA-seq and dRNA-seq data are from Canals *et al.* [20] and Hammarlöf *et al.* [21]. Each coloured horizontal track represents a different RNA-seq condition (Table S2), the upper panel shows sequence reads mapped to the positive strand and the lower panel reads mapped to the negative strand. The dRNA-seq data are shown in red, and were used to identify the TSSs, which are indicated by curved black arrows on the annotation track. Annotated phage genes are grouped into functional clusters. ncRNAs are annotated with red font. Striped arrows indicate pseudogenes and dotted red lines indicate where ORFs have been disrupted. Due to multiple copies of certain genes, some RNA-seq reads could not be mapped uniquely to the chromosome, these reads were ignored, and so transcriptomic signal is absent from parts of the prophage (e.g. the Gifsy-2 transposase *STMMW_10641*).

Unlike the BTP1 prophage transcriptome, which showed expression of the lytic genes in most conditions tested, the lytic genes of Gifsy-2 were not expressed (TPM≤10) (Fig. 2). The only highly-expressed genes (TPM>100) of Gifsy-2 were known prophage accessory genes, such as the virulence-associated genes *sodCI* (*STMMW_10551*), *gtgE* (*STMMW_10681*) and *sseI* (*STMMW_10631*). The *sseI* gene is a well-characterized pseudogene in strain D23580 generated by the insertion of a transposase [42]. Multiple copies of the inserted transposase gene (*STMMW_10641*) within the D23580 genome prevented the unique mapping of sequence reads, resulting in a blank region in the transcriptomic map (Fig. 2). The gene expression data for the Gifsy-2 prophage can be visualized interactively via the SalComD23580 online resource at https://tinyurl.com/SalComD23580-Gifsy2 and https://tinyurl.com/JBrowseD23580-Gifsy2.

Consistent with reports that Gifsy-2 is a lambdoid prophage [43], the Gifsy-2 homologue of the lambda CI repressor, *STMMW_10231*, was expressed (TPM>10) in all tested conditions. However, the induction behaviour of Gifsy prophages is distinct from lambda prophages. Gifsy prophages encode LexA-repressed antirepressor proteins, which, upon activation of the SOS response, inactivate the repressor protein [43]. This is unlike the inactivation of the repressor protein by activated RecA protein that occurs in lambda prophages. We observed transcription of the Gifsy-2 antirepressor gene, *gftA*, in most of our experimental conditions (Fig. 2), suggesting that additional regulatory mechanisms are involved in the induction in the Gifsy-2 prophage, such as post-transcriptional regulation of the *gftA* transcript.

To examine the conservation of gene expression patterns between prophages in different host backgrounds, the level of transcription of Gifsy-2 genes in the *S *. *enterica* serovar Typhimurium ST313 strain D23580 was compared to the level of transcription in the ST19 strain 4/74 [22] (Fig. S3). These two strains of *S *. *enterica* serovar Typhimurium belong to distinct multi-locus STs, and differ by 788 SNPs [20]. The most obvious difference between the expression patterns is that the Gifsy-2 ncRNA *IsrB-1* appeared to be expressed in strain 4/74, but not in D23580. However, previous investigation showed that *IsrB-1* is duplicated in the D23850 genome (being present on both Gifsy-2^D23580^ and Gifsy-1^D23580^ prophages) [20], meaning that *IsrB-1* RNA-seq reads could not be uniquely mapped to the D23580 genome. Therefore, the absence of *IsrB-1* expression in D23580 is an artefact, along with some other genetically redundant areas such as STMMW_10641 and a region overlapping STMMW_10591 to STMMW_10601. Aside from this discrepancy, a remarkable consistency in the gene expression of the Gifsy-2 prophage was observed between strains D23580 and 4/74, showing that prophage gene expression landscapes are independent of host background and highly reproducible between two phylogenetically distinct *Salmonella* strains.

#### Prophage ST64B

Previous studies have shown that induction of the ST64B prophage does not produce infectious phage particles due to a frameshift mutation in the tail assembly gene (*STMMW_19861–STMMW_19871*) [31, 44] (a mutation that is conserved in strain D23580). Virions released from induction of the defective ST64B prophage would lack a functional tail, preventing attachment to the bacterial surface and, therefore, making them refractory to plaque assay detection.

**Fig. 3.**
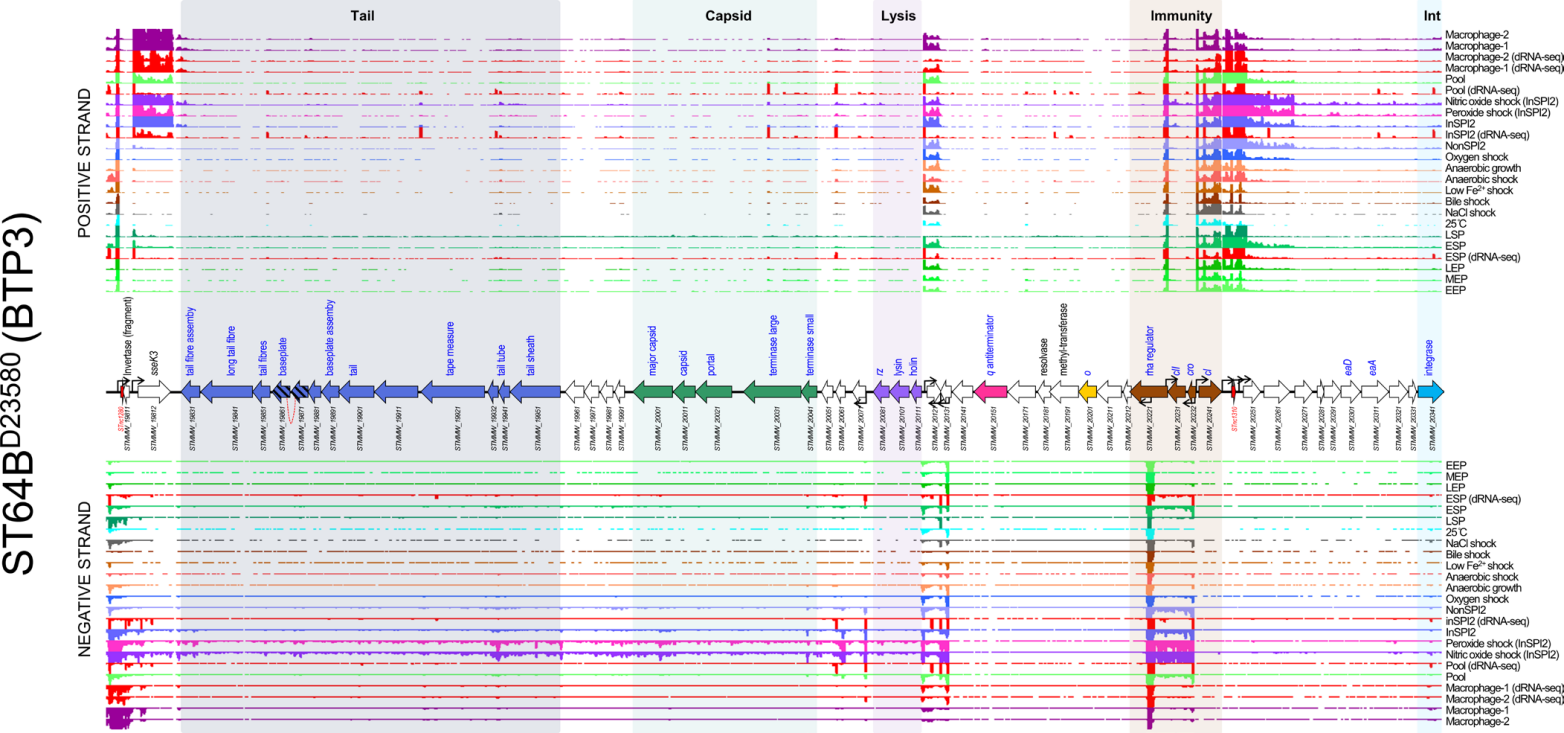
The transcriptomic landscape of the ST64B prophage of *S *. *enterica* serovar Typhimurium D23580 across 22 different RNA-seq experiments. RNA-seq and dRNA-seq data are from Canals *et al.* [20] and Hammarlöf *et al.* [21]. Each coloured horizontal track represents a different RNA-seq condition (Table S2), and the upper panel shows sequence reads mapped to the positive strand and the lower panel reads mapped to the negative strand. The dRNA-seq data are shown in red, and were used to identify the TSSs, which are indicated by curved black arrows on the annotation track. Annotated phage genes are grouped into functional clusters. ncRNAs are annotated with red font. Striped arrows indicate pseudogenes and dotted red lines indicate where ORFs have been disrupted.

The ST64B prophage transcriptome (Fig. 3) only showed significant lytic-gene transcription in two of the RNA-seq conditions tested: peroxide shock and nitric oxide shock. Because hydrogen peroxide and nitric oxide cause oxidative stress and DNA damage, transcription of the lytic genes in these conditions likely represents induction of the defective ST64B prophage. We speculate that the ST64B prophage of D23580 is uniquely sensitive to peroxide and nitric oxide stresses as there is no evidence for increased induction of the remaining D23580 prophages in these two conditions. The STnc1310 ncRNA, and two genes STMMW_20251 and STMMW_20261 that are encoded downstream of the prophage CI repressor, were also highly expressed in the hydrogen peroxide and nitric oxide shock conditions. However, these features are functionally uncharacterized; therefore, the biological role of their expression is unclear.

ST64B contains an accessory gene that encodes the secreted effector protein SseK3. The *sseK3* gene was only expressed in conditions linked to infection inside mammalian macrophages, such as InSPI2 (a medium designed to mimic the intra-macrophage environment), peroxide shock, nitric oxide shock and the intra-macrophage environment. A similar expression profile was observed for genes that encode other *Salmonella* pathogenicity island (SPI) 2-translocated effector proteins in *S *. *enterica* serovar Typhimurium strain 4/74 [34].

The patterns of gene expression of ST64B^D23580^ and ST64B^4/74^ showed many similarities (Fig. S4), though in 4/74 the prophage did not show lytic gene expression in the peroxide shock condition, suggesting that the prophage could exhibit distinct induction behaviour in strain 4/74. We speculate this difference could be explained by minor variations in the gene content between ST64B^D23580^ and ST64B^4/74^ (gene names displayed in red in Fig. S4 are unique to ST64B^D23580^).

Whilst the ST64B prophage in strain D23580 is not capable of forming infectious virions, induction of the prophage could still cause cell lysis, assuming that the function of phage lysis genes is independent of intact virion formation. Our finding of peroxide-inducible lysis genes may be relevant for other strains of *S *. *enterica* serovar Typhimurium that may harbour functional versions of the ST64B prophage. The gene expression data for the ST64B prophage can be visualized interactively via the SalComD23580 online resource at https://tinyurl.com/SalComD23580-ST64B and https://tinyurl.com/JBrowseD23580-ST64B.

#### Prophage Gifsy-1

**Fig. 4.**
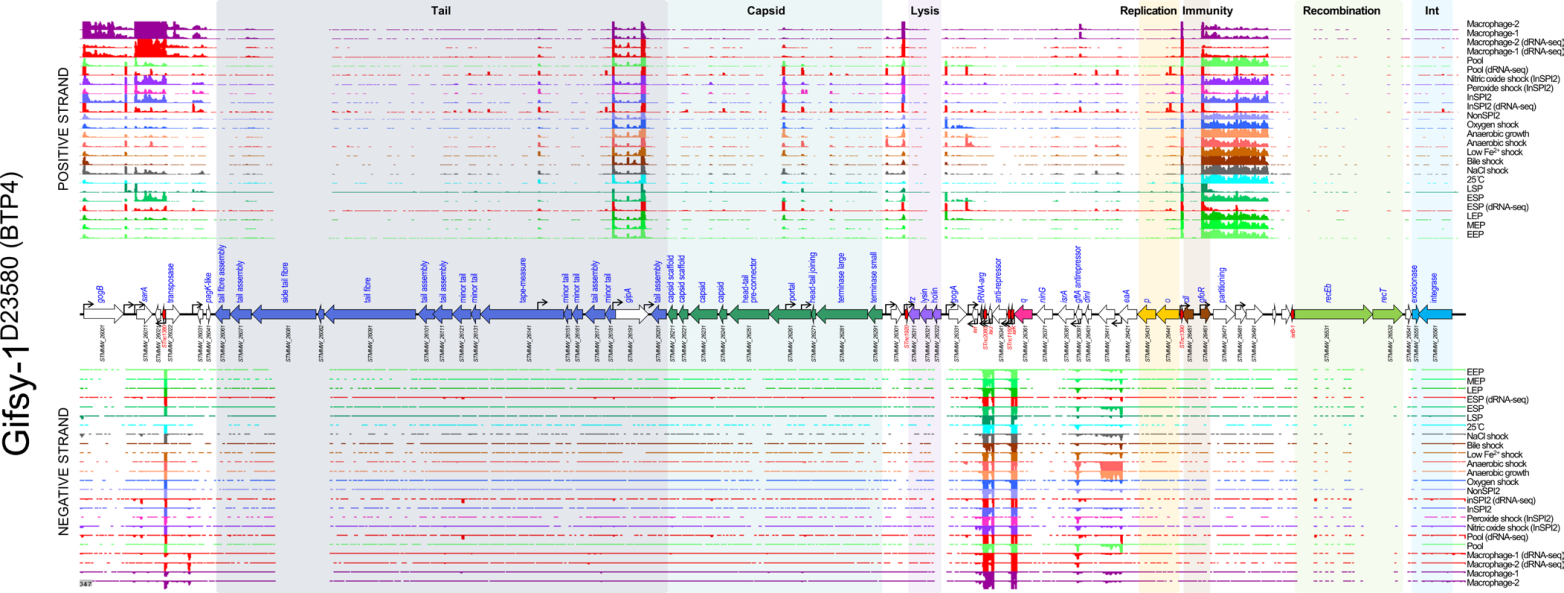
The transcriptomic landscape of the Gifsy-1 prophage of *S *. *enterica* serovar Typhimurium D23580 across 22 different RNA-seq experiments. RNA-seq and dRNA-seq data are from Canals *et al.* [20] and Hammarlöf *et al.* [21]. Each coloured horizontal track represents a different RNA-seq condition (Table S2), the upper panel shows sequence reads mapped to the positive strand and the lower panel reads mapped to the negative strand. The dRNA-seq data are shown in red, and were used to identify the TSSs, which are indicated by curved black arrows on the annotation track. Annotated phage genes are grouped into functional clusters. ncRNAs are annotated with red font.

Like the Gifsy-2^D23580^ prophage, there was no evidence of lytic-gene transcription of Gifsy-1^D23580^ in any of the 17 environmental conditions examined (Fig. 4). This is consistent with the very low level of spontaneous induction observed for the resuscitated Gifsy-1 prophage in our previous study [31]. Gifsy-1^D23580^ expressed a number of ncRNAs that were highly transcribed in all conditions tested, including STnc1380, STnc2080, IsrJ, STnc1160 and IsrK [45]. The virulence-associated genes *gogB* (*STMMW_26001*), *steE* (*STMMW_26011*, previously known as *pagJ* and *sarA*) and *pagK* (*STMMW_26041*) were only expressed in intra-macrophage infection-related conditions. In contrast, the virulence-associated gene *gipA* (*STMMW_26191*) was transcribed in all conditions tested, despite being previously reported to be specifically induced during colonization of the small intestine [46]. Another virulence-associated gene, *gogA* (*STMMW_26331*), showed very little transcription in any of the conditions studied. The functionally uncharacterized gene *STMMW_26411* was specifically induced in the anaerobic shock and anaerobic growth conditions, suggesting that this gene could play a role when the bacterium experiences absence of oxygen.

The Gifsy-1 prophage is not identical between strains D23580 and 4/74, and shows considerable difference in gene content, particularly at the 3′ proximal end. Therefore, comparison of the Gifsy-1 gene expression profiles between the two strains is difficult to interpret (Fig. S5). However, the expression patterns of orthologous genes shared by the two prophages were similar across the multiple conditions. The gene expression data for the Gifsy-1 prophage can be visualized interactively via the SalComD23580 online resource at https://tinyurl.com/SalComD23580-Gifsy1 and https://tinyurl.com/JBrowseD23580-Gifsy1.

#### Prophage BTP5

**Fig. 5.**
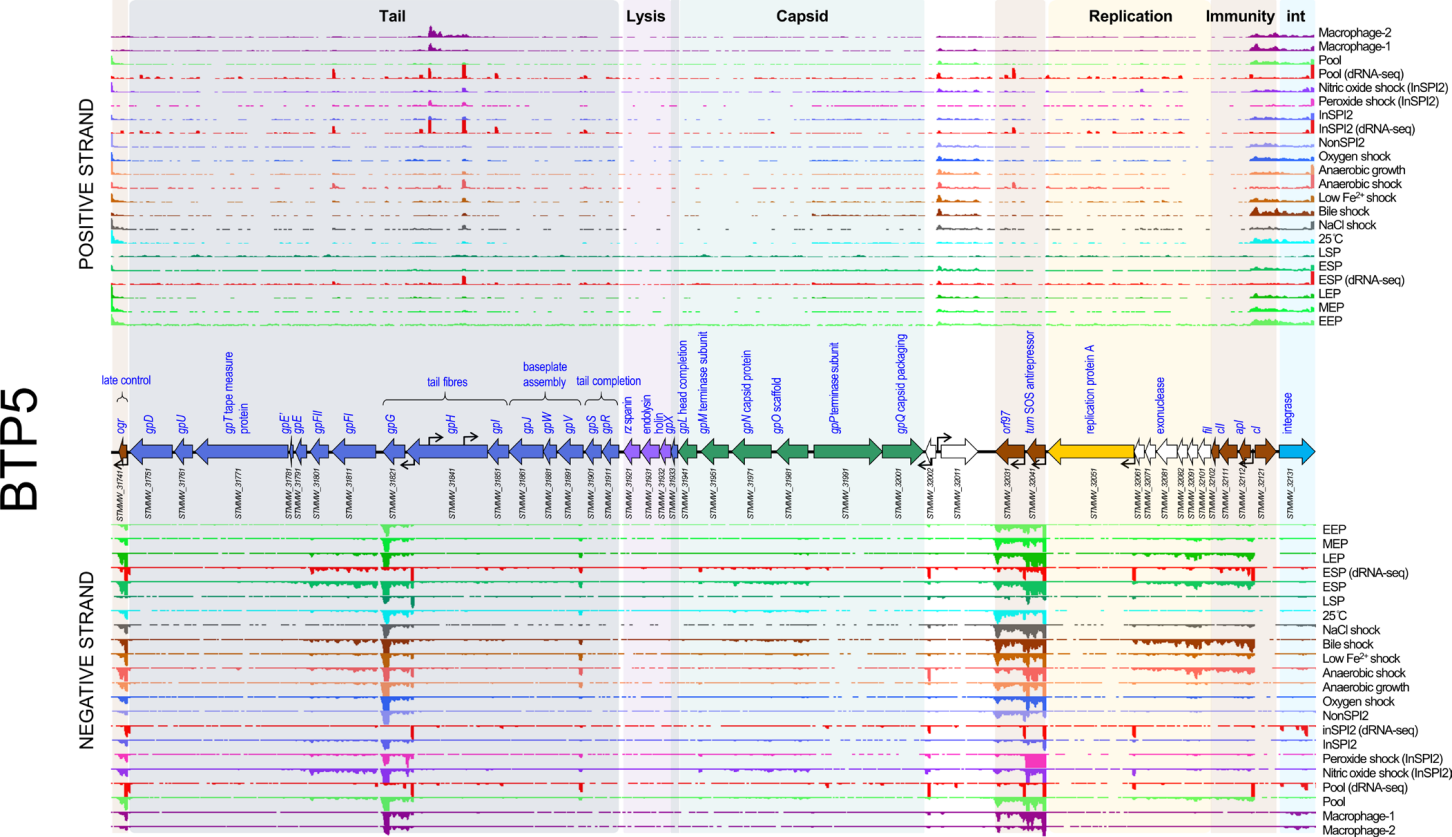
The transcriptomic landscape of the BTP5 prophage of *S *. *enterica* serovar Typhimurium D23580 across 22 different RNA-seq experiments. RNA-seq and dRNA-seq data are from Canals *et al.* [20] and Hammarlöf *et al.* [21]. Each coloured horizontal track represents a different RNA-seq condition (Table S2), and the upper panel shows sequence reads mapped to the positive strand and the lower panel reads mapped to the negative strand. The dRNA-seq data are shown in red, and were used to identify the TSSs, which are indicated by curved black arrows on the annotation track. Annotated phage genes are grouped into functional clusters.

The BTP5 prophage was the least transcriptionally active of all the D23580 prophages (Figs 5 and S5). However, the expression pattern of BTP5 genes does not provide insight into the biology of the prophage. The most highly expressed transcript in the prophage belonged to the *tum* gene (*STMMW_32041*), a homologue of the Tum antirepressor of coliphage 186 [47]. The antirepressor was expressed at high level particularly in the nitric oxide shock condition, raising the possibility that nitric oxide could stimulate induction of BTP5. We previously showed that infectious BTP5 phages could not be detected from strain D23580, with or without chemical induction with mitomycin C [31], but the apparent activation of the antirepressor in response to hydrogen peroxide stress is consistent with a specific induction behaviour of the BTP5 prophage. Alternatively, as suggested for the anti-repressor of prophage Gifsy-2, the BTP5 antirepressor may be regulated at the post-transcriptional level.

In BTP5, transcription was observed from the promoter upstream of the *apI* gene (*STMMW_32112*) through to the uncharacterized gene *STMMW_32601* in certain conditions, including early stationary phase (ESP), bile shock and anaerobic shock. The corresponding genes in coliphage 186 belong to the early lytic operon [48] and represent the genes initially expressed during lytic phage replication. Transcription of a three-gene operon consisting of tail structural genes (*STMMW_31821*, *STMMW_31811* and *STMMW_31801*) was observed in a number of conditions and was particularly high in ESP, bile shock and nitric oxide shock. The tail structure of P2-like phages (Myoviruses) is complex [49], and expression of these three genes alone would not produce functional phage tail particles, making the functional relevance of this transcript unclear. Additionally, the *ogr* gene (*STMMW_31741*), reported to be involved in control of late gene expression in phage P2 [49], was expressed in all conditions tested.

Despite the transcription of a subset of lytic genes in the BTP5 prophage, the repressor and integrase genes were transcribed in all conditions examined, albeit at low levels relative to the level of tail gene transcripts. We conclude that the BTP5 prophage transcriptome does not inform the functionality of the prophage and, consistent with our previous study, the lysogeny and lysis behaviour of the BTP5 prophage remains enigmatic [31]. We speculate that there may be further control of prophage BTP5 gene expression at the post-transcriptional level, or alternatively the transcriptome may reflect heterogeneity in the activity of the BTP5 prophage across the bacterial cell population. The gene expression data for the BTP5 prophage can be visualized interactively via the SalComD23580 online resource at https://tinyurl.com/SalComD2358-BTP5 and https://tinyurl.com/JBrowseD23580-BTP5.

### Characterized prophage accessory loci have unique transcriptional signatures

**Fig. 6.**
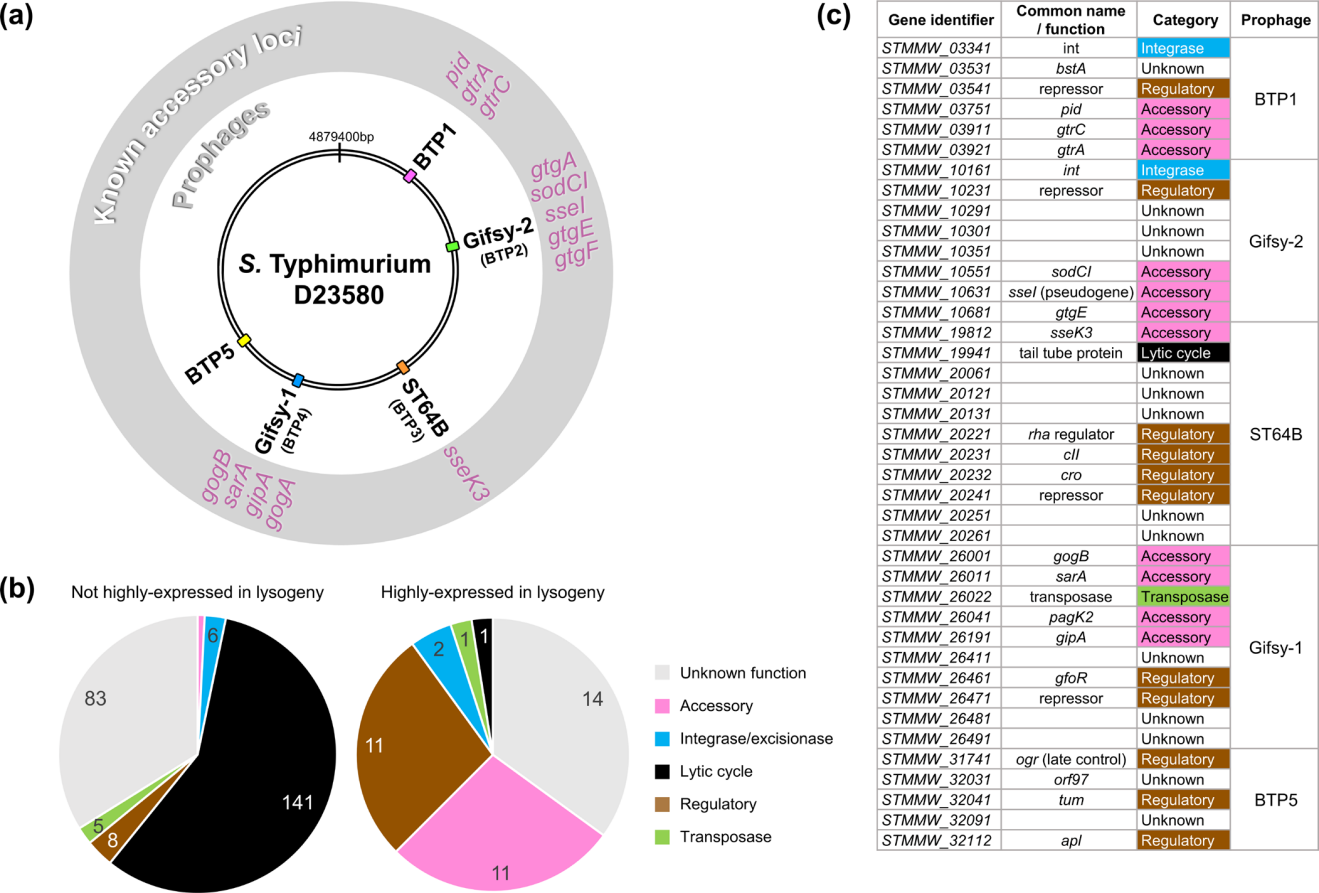
Prophage regulatory or accessory genes show unique transcriptional signatures. These findings suggest that transcriptomic data can be used to heuristically enrich for genes likely to be associated with novel regulatory or accessory functions. (a) Genomic map of *S *. *enterica* serovar Typhimurium strain D23580 indicating the location of the five prophage elements. Known accessory loci associated with each prophage element are annotated in the exterior grey ring. (b) Functional categorization of all prophage genes with expression values of <100 TPM (not highly expressed in lysogeny) and >100 TPM (highly expressed in lysogeny) in at least one RNA-seq condition. The majority of highly expressed prophage genes have a known regulatory or accessory function, or have no known function. (c) The 40 prophage genes of *S *. *enterica* serovar Typhimurium strain D23580 classified as highly expressed during lysogeny.

*S *. *enterica* serovar Typhimurium prophages BTP1, Gifsy-2^D23580^, ST64B^D23580^ and Gifsy-1^D23580^ contain characterized accessory loci, including genes involved in *Salmonella* pathogenicity or phage exclusion (Fig. 6a). To empirically determine whether known prophage accessory loci had distinct transcriptional signatures compared with the rest of the prophage, we used an expression cut-off of 100 TPM [20] to identify highly expressed genes. Genes with expression values of >100 TPM in at least one RNA-seq experiment were classified as highly expressed during lysogeny (Table S5), and were assigned to one of the following functional categories based on annotation: unknown function, accessory gene, regulatory gene, integrase, transposase or structural gene (Fig. 6b). Of the 278 genes annotated in the five prophages, 40 genes (14%) (Fig. 6c) were highly expressed during lysogeny. As expected, many genes in the highly expressed category represented known accessory genes (11 genes), such as genes encoding type three secretion system (T3SS) effectors, or regulators (11 genes) including repressors. Among the other highly expressed genes were genes encoding one transposase, one prophage structural protein and two prophage integrases. However, the largest category of highly expressed genes were those of unknown function (14 genes) [50]. We conclude that the transcriptional signatures are consistent with these 14 genes being novel prophage accessory genes or regulatory genes. We note that this ‘guilt by association’ approach has previously been successfully used to identify novel SPI-regulated genes [22], and to make broader regulatory deductions [51, 52].

### Identification of candidate novel prophage regulatory or accessory loci

**Fig. 7.**
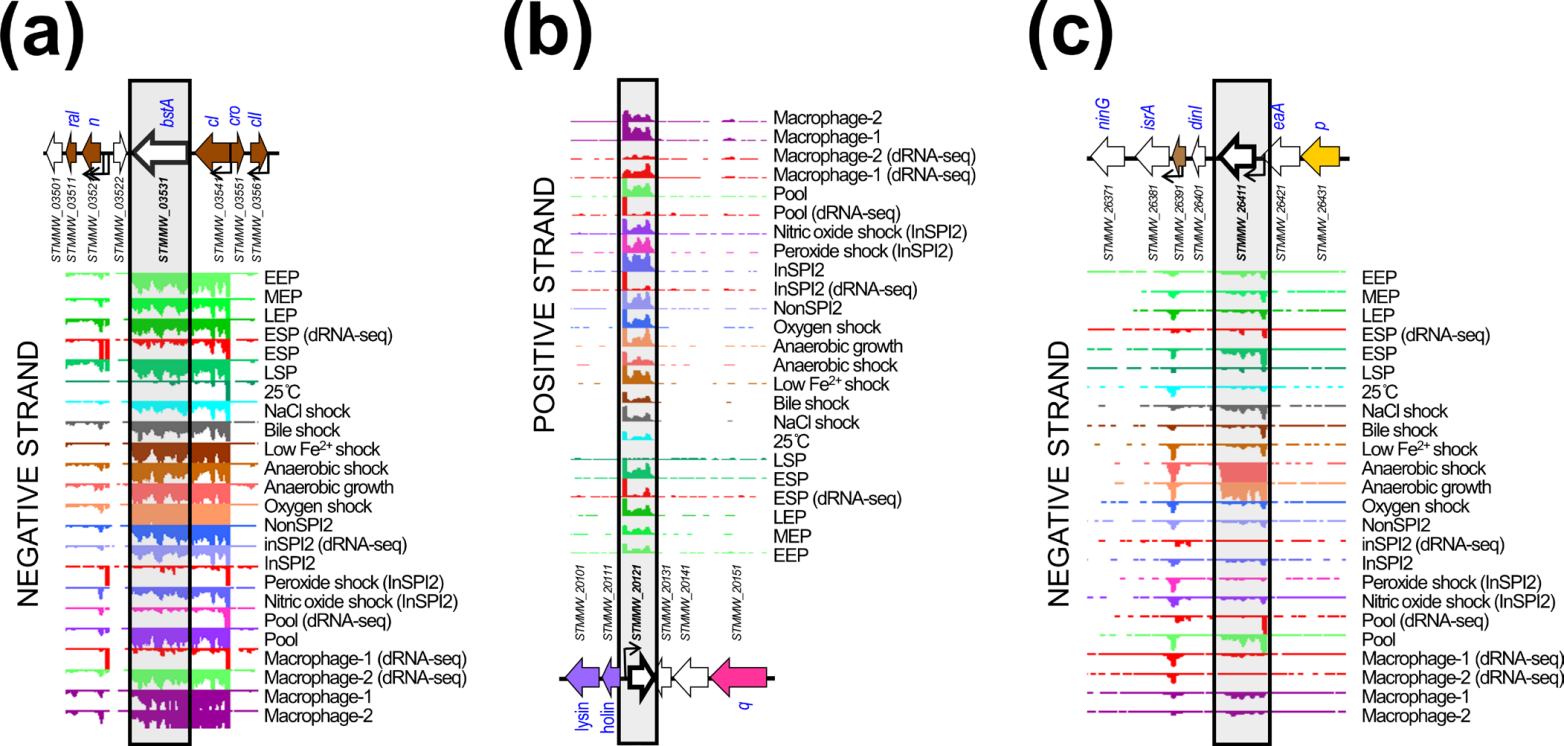
Examples of prophage genes exhibiting unique transcriptional signatures consistent with regulatory or accessory functions. (a) The *bstA* locus of prophage BTP1. (b) The *STMMW_20121* locus of prophage ST64B. (c) The *STMMW_26411* locus of prophage Gifsy-1.

Fig. 7 shows the genomic and transcriptomic context of three of the prophage genes of unknown function identified in this study and likely to represent novel prophage regulatory or accessory loci. The *bstA* locus of prophage BTP1 lies down-stream of the *cI* repressor locus, and is transcribed from the promoter of the *cI* gene in all the 17 environmental conditions (Fig. 7a). The region between the repressor locus and the *n* locus of lambdoid prophages has been previously shown to have a high frequency of mosaicism and represents a common site of prophage accessory (moron) loci, such as the *rexAB* locus of phage lambda [53]. The *bstA* gene (*STMMW_03531*, formerly designated *ST313-td*) has been implicated as a determinant of both virulence [54] and anti-virulence [55] in *S. enterica* strains, though no mechanism for these phenotypes has been proposed (hence, our conservative inclusion of *bstA* in the ‘unknown function’ functional category in this study). Our transcriptomic data support a functional role for *bstA* as a novel prophage accessory gene that may modulate the biology of the lysogen, through interaction with bacterial physiology or with other prophages present in the genome.

Like *bstA*, the *STMMW_20121* locus (Fig. 7b) of prophage ST64B^D23580^ showed transcription in almost all infection-relevant conditions included in our study. However, we observed that *STMMW_20121* was independently transcribed from its own promoter. *STMMW_20121* is encoded antisense to the prophage lytic genes, a region that is characteristic of prophage accessory (moron) loci [7]. The transcriptomic signature and genomic context of *STMMW_20121* is consistent with the gene product having an accessory or regulatory function.

Finally, we identified the *STMMW_26411* locus (Fig. 7c) of prophage Gifsy-1^D23580^ as a candidate prophage accessory gene. Unlike *bstA* and *STMMW_20121*, *STMMW_26411* showed highly condition-specific transcription, associated with anaerobic conditions. The environmental specificity of *STMMW_26411* transcription leads us to speculate that the gene is more likely to be a novel accessory gene than function as a prophage regulatory gene, particularly given the lack of corresponding lytic gene transcription in the Gifsy-1^D23580^ prophage under anaerobic conditions. It is possible that the function of *STMMW_26411* is linked to the facultative anaerobic lifestyle of *S *. *enterica* serovar Typhimurium and the ability of the pathogen to colonize the mammalian gastrointestinal tract [56]. Given the broad conservation of the Gifsy-1 prophage amongst *S *. *enterica* serovar Typhimurium strains [18] and known association of this prophage with virulence factors (Fig. 6a), the *STMMW_26411* locus represents an exciting candidate accessory factor for further study.

### Identification of a novel prophage-encoded ncRNA involved in superinfection exclusion

**Fig. 8.**
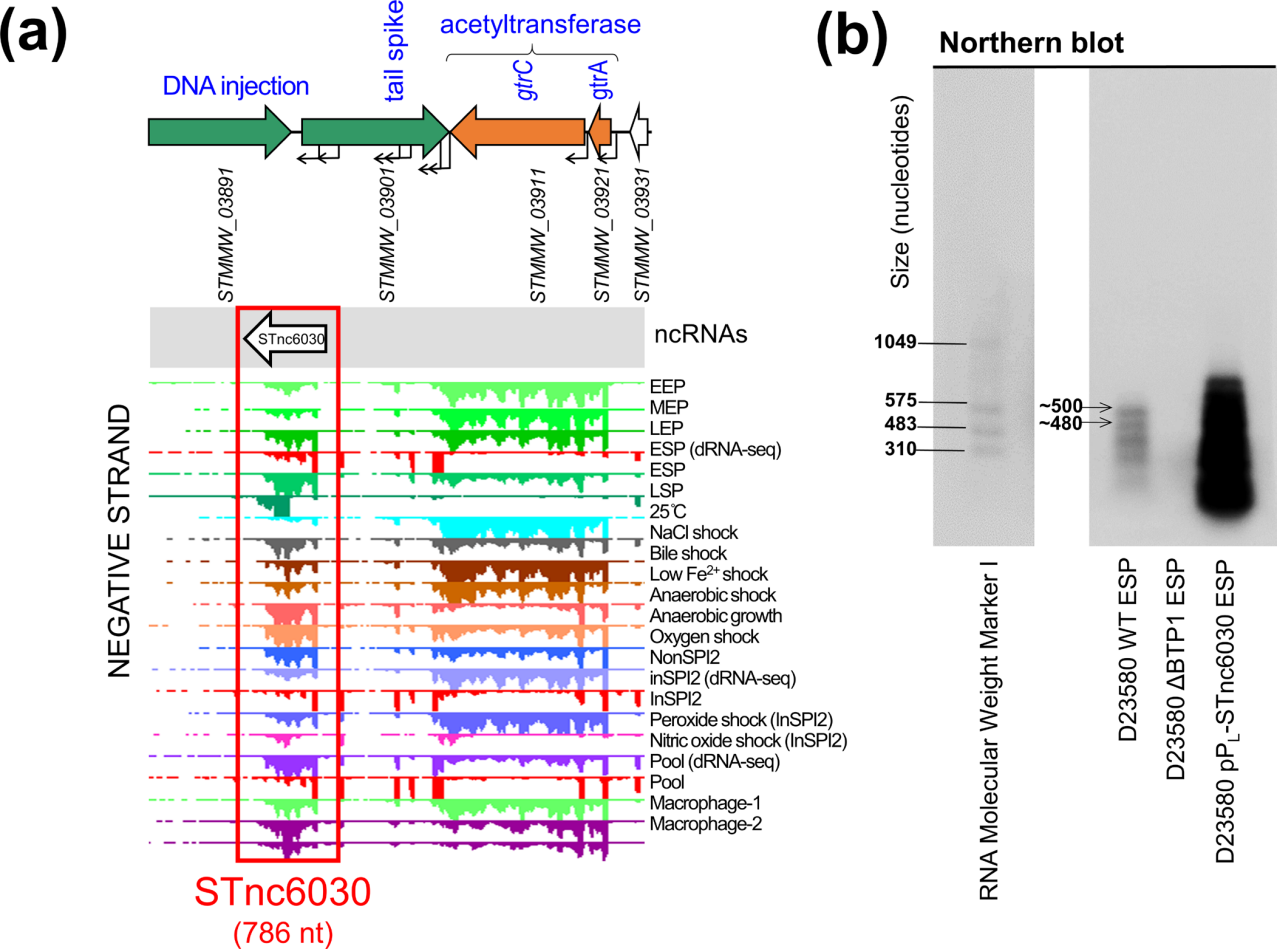
Transcriptomic-based identification of a novel prophage-encoded asRNA, STnc6030. (a) The transcriptional context of the STnc6030 ncRNA. The transcriptomic data are the same as shown in Fig. 1. (b) Detection of the STnc6030 transcript by Northern blot using an anti-STnc6030 DIG-labelled riboprobe. The two most abundant transcripts detected by the anti-STnc6030 riboprobe are indicated, with the approximate size estimated from the molecular marker.

As well as identifying novel candidate prophage accessory and regulatory genes, our transcriptomic analysis of the prophages of D23580 revealed a number of putative novel ncRNAs. We focused on the ncRNAs of prophage BTP1 (Fig. 1), as this prophage is functional [31], yet poorly characterized. To identify the biological relevance of novel ncRNAs, STnc6030 was selected for further investigation as it was particularly highly expressed in the majority of infection-relevant growth conditions (Fig. 8a). Additionally, the putative ncRNA is unusually long, >700 nt in length, and is positioned antisense to the BTP1 tailspike gene (*STMMW_03901*) and a gene encoding a putative DNA injection protein (*STMMW_03891*). It should be noted that the STnc6030 region is an area of unusually high transcription, with seven sense and antisense TSSs defined within the tailspike gene alone.

The length and antisense location of the STnc6030 transcript led us to hypothesize that the putative ncRNA was an asRNA species. The majority of characterized bacterial asRNAs function as inhibitors of target RNA function [57], and are commonly found in accessory genome elements such as phages and plasmids [58]. We hypothesized that STnc6030 interacts with the transcription of the BTP1 tailspike gene, and investigated this experimentally. Fig. 8(a) shows a detailed view of STnc6030 transcription within the BTP1 transcriptome. Analysis of the STnc6030 transcript region in all three reading frames did not reveal any ORFs >60 amino acids in length, supporting the classification of STnc6030 as an ncRNA species. The beginning of the STnc6030 transcript corresponds to the beginning of RNA-seq reads mapping to the tailspike gene on the sense strand, consistent with antisense interference with the tailspike gene transcript, which can be visualized at https://tinyurl.com/BTP1-tailspike-TSS.

The sequence of the putative STnc6030 asRNA was derived from the BTP1 transcriptomic data, and was cloned into the pP_L_ expression plasmid under the control of the constitutive P_LlacO-1_ promoter. To confirm the presence of the STnc6030 transcript, an anti-STnc6030 riboprobe was synthesized to detect the STnc6030 RNA species by Northern blot (Fig. 8b). The riboprobe was designed to cover the whole of the approximately 786 nt STnc6030 transcript, allowing the detection of any transcripts corresponding to this region. The anti-STnc6030 riboprobe detected a number of transcripts in the D23580 WT strain, whilst no transcripts were detected in the D23580 ΔBTP1 mutant, confirming the specificity of detected bands. The largest transcript detected by the anti-STnc6030 probe in the D23580 WT was approximately 500 nt in length, significantly shorter than the predicted length of 786 nt. At least two other smaller transcripts were detected, of ~500 and ~480 nt in length, which could result from RNA processing or degradation products, as seen for other *Salmonella* ncRNAs such as ArcZ [59].

**Fig. 9.**
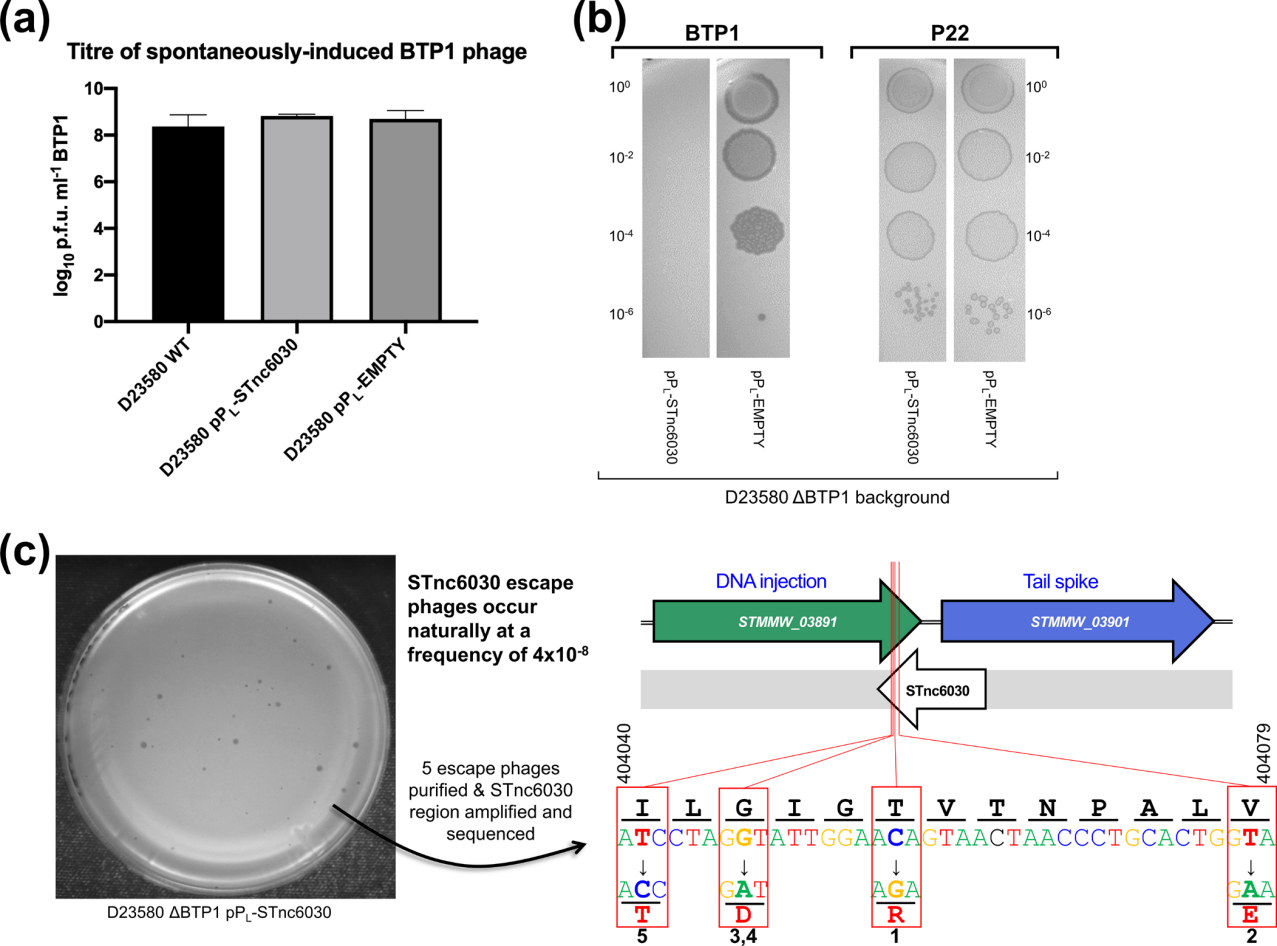
The BTP1 prophage-encoded asRNA STnc6030 functions as a phage-specific superinfection-exclusion factor. (a) Over-expression of the STnc6030 RNA in D23580 WT background does not affect BTP1 spontaneous induction. Plaque assay of overnight culture supernatants of D23580 WT, D23580 pP_L_-STnc6030 and D23580 pP_L_ (empty vector) on host strain D23580 ΔBTP1. Error bars represent the sd of three biological replicates. (b) Heterologous expression of STnc6030 in D23580 ΔBTP1 completely protects against BTP1 phage, but not P22 infection. Plaque assay of BTP1 and P22 phage on D23580 ΔBTP1 strains containing the pP_L_-STnc6030 expression plasmid or the negative control plasmid. (c) Isolation of STnc6030-escape mutants of phage BTP1 suggests the STnc6030 functional ‘seed’ region is located at the 3’ end of the transcript. A high titre BTP1 phage stock was used to identify naturally occurring BTP1 phage mutants that were immune to inhibition by STnc6030. Escape phages were estimated to occur at a frequency of approximately 4×10^−8^. Five escape phages of varying plaque morphologies were selected for sequencing. The sequence of the STnc6030 region of the escape phages identified SNPs, and the position and substitution are shown. The SNPs that conferred immunity to STnc603*0* interference were clustered within a 36 bp region (shown) corresponding to the 3’ end of the STnc6030 transcript and the 3’ end of the *STMMW_03891* gene (located between nt 404 041 and 404 078 on the D23580 chromosome; GenBank accession no. FN424405).

To interrogate the biological function of STnc6030, D23580 WT and D23580 ΔBTP1 (sensitive to phage BTP1) were transformed with the pP_L_-STnc6030 and empty vector control plasmids. The BTP1 prophage displays an unusually high level of spontaneous induction in the lysogenic cell population [31] and we hypothesized that STnc6030 may contribute to this phenotype. However, over-expression of STnc6030 RNA did not affect the level of BTP1 spontaneous induction in the D23580 WT background, and no difference in the number of spontaneously induced BTP1 phage was observed in overnight culture supernatants of D23580 WT, D23580 pP_L_-STnc6030 and D23580 pP_L_ (empty vector) (Fig. 9a).

Next, we investigated whether STnc6030 could play a role in phage immunity. Expression of STnc6030 in a naïve host in the absence of the BTP1 prophage (D23580 ΔBTP1 pP_L_-STnc6030) mediated total immunity to BTP1 infection (Fig. 9b), but did not modulate susceptibility to phage P22 infection. These results were consistent with a regulatory mechanism in which STnc6030 targeted the sense transcript of the BTP1 DNA injection and tailspike genes for degradation by base pairing. RNA–RNA interactions require high nucleotide complementarity, and consistent with this model, the corresponding region of the P22 genome does not share similarity to BTP1 at the nucleotide level.

To explore the functionality of the STnc6030 transcript, a high titre BTP1 phage stock was screened for the presence of naturally occurring mutants resistant to exclusion by STnc6030 (Fig. 9c). The BTP1 phage stock was plated on lawns of D23580 ΔBTP1 containing the pP_L_-STnc6030 expression plasmid, escape phages arose at a frequency of 4×10^−8^ and had varying plaque sizes (Fig. 9c). Five escape phages were isolated and purified, and a nested PCR strategy was used to amplify the STnc6030 transcript region of the escape phages. Sequencing of the STnc6030 region revealed that each escape phage contained a SNP relative to the BTP1 WT sequence (Fig. 9c). In total, four unique SNPs were identified that conferred resistance to STnc6030-mediated exclusion. The four SNPs clustered within a 36 bp region corresponding to the 3′ end of the STnc6030 transcript, antisense to the putative DNA injection gene *STMMW_03891* (Fig. 9c). These data implicate the 3′ end of STnc6030 as a functionally active ‘seed’ region of the RNA that interacts with the antisense target (*STMMW_03891*). The four SNPs also cause non-synonymous amino acid substitutions to the STMMW_03891 protein (Fig. 9c).

Together, these results are consistent with a model where the STnc6030 asRNA acts as a superinfection-exclusion factor, inhibiting re-infection of the BTP1 phage lysogen with ‘self’ phage, or preventing the infection of closely related phages that share sequence identify to BTP1 in the STnc6030 region. However, the fact that the BTP1 prophage induction can occur normally in the presence of the STnc6030 asRNA (Fig. 9a) suggests that the induced BTP1 prophage has a mechanism to escape the effects of its own superinfection-exclusion RNA. Further work is required to confirm the biological activity of the STnc6030 asRNA of the BTP1 prophage. Our data illustrate the power of transcriptomic data for uncovering novel prophage biology, particularly because novel ncRNA species can only be identified at the transcriptional level, and not by comparative genomic analysis.

## Discussion

Prophage accessory loci can mediate the lysogenic conversion of the bacterial host, by encoding virulence factors or superinfection immunity factors [9]. A study in *Pseudomonas aeruginosa* showed that 12 out of 14 previously uncharacterized accessory (moron) loci affected diverse bacterial phenotypes, including phage immunity, motility and biofilm formation [60]. However, prophage accessory genes are difficult to identify from DNA sequence alone. We reasoned that prophage accessory loci were likely to be associated with unique transcriptional signatures compared to phage lytic genes, because in order to affect the biology of the host cell, they must be expressed during lysogeny. Here, we show that transcriptomic data, pre-existing or purposefully generated, provide unique insights into the molecular biology of prophages, and we propose that transcriptional signatures will improve our ability to identify and annotate prophage regulatory and accessory genes.

Inferences from the transcriptomes of the D23580 prophage regions were generally consistent with our previous findings concerning the functionality of the prophages [31]. BTP1, a prophage that exhibits a high level of spontaneous induction, showed an unusually high level of transcriptional activity for a prophage region. Canonically, prophage genes are expected to be transcriptionally repressed during lysogeny, apart from those genes required to maintain prophage lysogeny, such as the gene encoding the CI repressor in lambdoid prophages [1] or genes with accessory functions that confer a fitness advantage to the lysogen.

Unlike the four other prophage regions of D23580, low-level transcription of the BTP1 structural genes was observed in almost all growth conditions tested. A lysogenic cell cannot constitutively express phage structural and lytic genes, because once the prophage molecular switch has moved to lytic from lysogenic replication, the unavoidable consequence is cell death [1]. Therefore, if the observed lytic gene expression occurred in the entire cellular population, a population collapse would ensue, as ultimately sufficient lysis proteins were accumulated to initiate cell lysis. However, the BTP1 prophage lysogen (D23580) exhibits normal growth dynamics that are comparable to strains not lysogenized by the BTP1 prophage [31]. We propose that the lytic gene expression observed in our transcriptomic data reflects the unavoidable averaging of gene expression across a heterogeneous bacterial population in which lytic genes are highly expressed in the approximately 0.2 % of the cells that undergo spontaneous BTP1 prophage induction [31].

Consistent with this model, the remaining D23580 prophages (Gifsy-2^D23580^, ST64B^D23580^, Gifsy-1^D23580^ and BTP5) do not exhibit significant spontaneous induction levels [31] and show little lytic gene expression in the majority of growth conditions (Figs 2–5). The only other D23580 prophage to show evidence of late gene transcription was ST64B^D23580^ in two growth conditions (peroxide and nitric oxide stress), which may reflect specific induction behaviour of the ST64B^D23580^ prophage. In light of this finding, we speculate that the absolute expression levels of prophage structural genes could be used to estimate the fraction of the lysogenic population undergoing lytic prophage replication.

As well as providing insight into the replication state of the D23580 prophages, the transcriptome maps also allow the identification of putative accessory regions, genes or transcripts expressed during lysogenic replication, or novel genes involved in the regulation of lysogeny. Prophage accessory genes are of importance for bacterial pathogens, as they could be ‘smoking guns’ responsible for rapid changes in disease tropism. Prophages BTP1 and BTP5 are specific to the epidemic African ST of *S *. *enterica* serovar Typhimurium ST313, and have not yet been well characterized.

The transcriptome maps of prophages BTP1 and BTP5 showed several more regions of transcription than are theoretically necessary for a lambdoid prophage to maintain lysogeny (typically the CI repressor only) [61]. A number of genes in the BTP1 prophage showed an expression pattern consistent with an accessory or regulatory function, including *bstA*, *pid*, *gtrA^BTP1^* and *gtrC^BTP1^*. Of these four genes, all except *bstA* have mechanistically described accessory functions. Prophages Gifsy-2, ST64B and Gifsy-1 are broadly conserved in many strains of *S. enterica* [18] and encode numerous virulence genes including T3SS effector genes [62]. Although the regulatory behaviour and accessory genes of these prophages have been studied for decades, we identified numerous novel candidate accessory and regulatory genes, demonstrating the power of our gene expression profiling approach.

Whilst RNA-seq-based transcriptomics represents a useful tool for discovering coding gene functions, it is arguably an even more powerful approach for the discovery of non-coding genomic elements such as ncRNAs [63]. A number of putative RNA transcripts in the BTP1 prophage that did not correspond to protein coding sequences had expression profiles consistent with an accessory function, including eight putative novel ncRNAs [20]. Several prophage-encoded ncRNAs have been implicated in bacterial virulence, for example, the Gifsy-1 prophage encoded IsrJ, an approximately 74 nt ncRNA required for efficient invasion of *Salmonella* into nonphagocytic cells and effector translocation by the SPI-1 T3SS [45]. Prophage-encoded ncRNAs also mediate non-virulence accessory functions, including the *sas* asRNA of phage P22 that induces a translational switch between distinct peptides encoded by the *sieB* gene, and is critical to the function of the SieB superinfection-exclusion system [64]. Lastly, the phage λ ncRNA OOP inhibits CII protein synthesis, thereby pushing the phage molecular decision towards lysis, rather than lysogeny [40].

Our transcriptomic approach identified the STnc6030 asRNA encoded within the BTP1 prophage late genes. The transcript is located antisense to the 3′ end of the putative DNA-injection gene *STMMW_03891* and 5′ end of the tailspike gene *STMMW_03901*. Expression of STnc6030 in a heterologous host abolished susceptibility to infection by BTP1, but not to the related phage P22. However, the spontaneously induced titre of BTP1 phage was not affected by overexpression of the STnc6030 transcript, suggesting that the asRNA does not interfere with replication of spontaneously induced BTP1.

Bacterial asRNAs, also known as *cis*-encoded RNAs, are usually found antisense to annotated coding genes. The extensive genetic complementarity with the corresponding transcripts allows asRNAs to affect the stability of complementary mRNA transcripts by base-pair interactions [65]. Because dsRNA molecules are substrates for endoribonucleases, the effect of asRNA targeting is usually to increase the degradation of particular mRNA transcripts and so reduce levels of the gene product. Alternatively, base-pairing of two RNA species can block a ribonuclease recognition site, leading to increased stability of the target mRNA [58].

Our data are consistent with a model where the functional mechanism of the STnc6030 asRNA is a base-pairing interaction with the transcript containing the DNA-injection gene *STMMW_03891*, with a concomitant decrease in the stability of the mRNA. As prophage genes are frequently expressed as polycistronic operons encoding multiple genes required by the replicating phage, the antisense targeting of a single prophage gene could destabilize a long transcript encoding the entirety of the prophage lysis and structural genes, effectively inhibiting prophage replication. However, it remains unclear how the STnc6030 asRNA, which is natively located within BTP1, avoids interference with the BTP1 prophage upon induction from lysogenic growth within the bacterial chromosome. The mechanism by which the induced BTP1 prophage escapes its own immunity asRNA remains to be discovered. The BTP1 prophage encodes two other systems for superinfection exclusion, the GtrAC^BTP1^ system and the CI^BTP1^ repressor [38], and the precise biological role of the STnc6030 asRNA in the context of these other systems requires further study.

Overall, our work represents what is, to the best of our knowledge, the first detailed report of the transcriptional landscapes of native bacterial prophages. A wealth of RNA-sequencing data exists for a number of poorly characterized bacterial pathogens in which virulence factors and prophage molecular regulation have not been characterized. As well as identifying novel candidate regulatory and accessory loci in *Salmonella* prophages, our work represents a ‘proof of concept’ study that shows that careful analysis of RNA-seq data mapped to prophage regions could reveal a vast array of novel prophage accessory loci. Prophage transcriptomic maps represent a powerful window through which to view the molecular biology of temperate phages.

## Data bibliography

1. Owen SV, Canals R, Wenner N, Hammarlöf DL, Kröger C, Hinton JCD. All the RNA-seq and genome data used in this study are publicly available and can be accessed using the accession numbers in Table S1 (2020).

2. The RNA-seq data that underpin this study are available for interrogation as searchable gene expression profiles at https://tinyurl.com/SalComD23580, and can be visualized in the context of a genome browser e.g. at https://tinyurl.com/BTP1-tailspike-TSS. (2020)

3. Perez-Sepulveda BM, Hinton JCD. Functional transcriptomics for bacterial gene detectives. *Microbiol Spectr* 2018;6:RWR-0033-2018. Our recent review article describing strategies for using the SalCom databases as a discovery tool (2018).

## Supplementary Data

Supplementary material 1

Supplementary material 2
