# Supplementary material for: A window into lysogeny: revealing temperate phage biology with transcriptomics
Source: Microb Genom. 2020 Feb 5;6(2):e000330. doi: 10.1099/mgen.0.000330 (PMC7067206; doi:10.1099/mgen.0.000330)
Supplement: Supplementary material 2 [file mgen-6-330-s002.pdf]

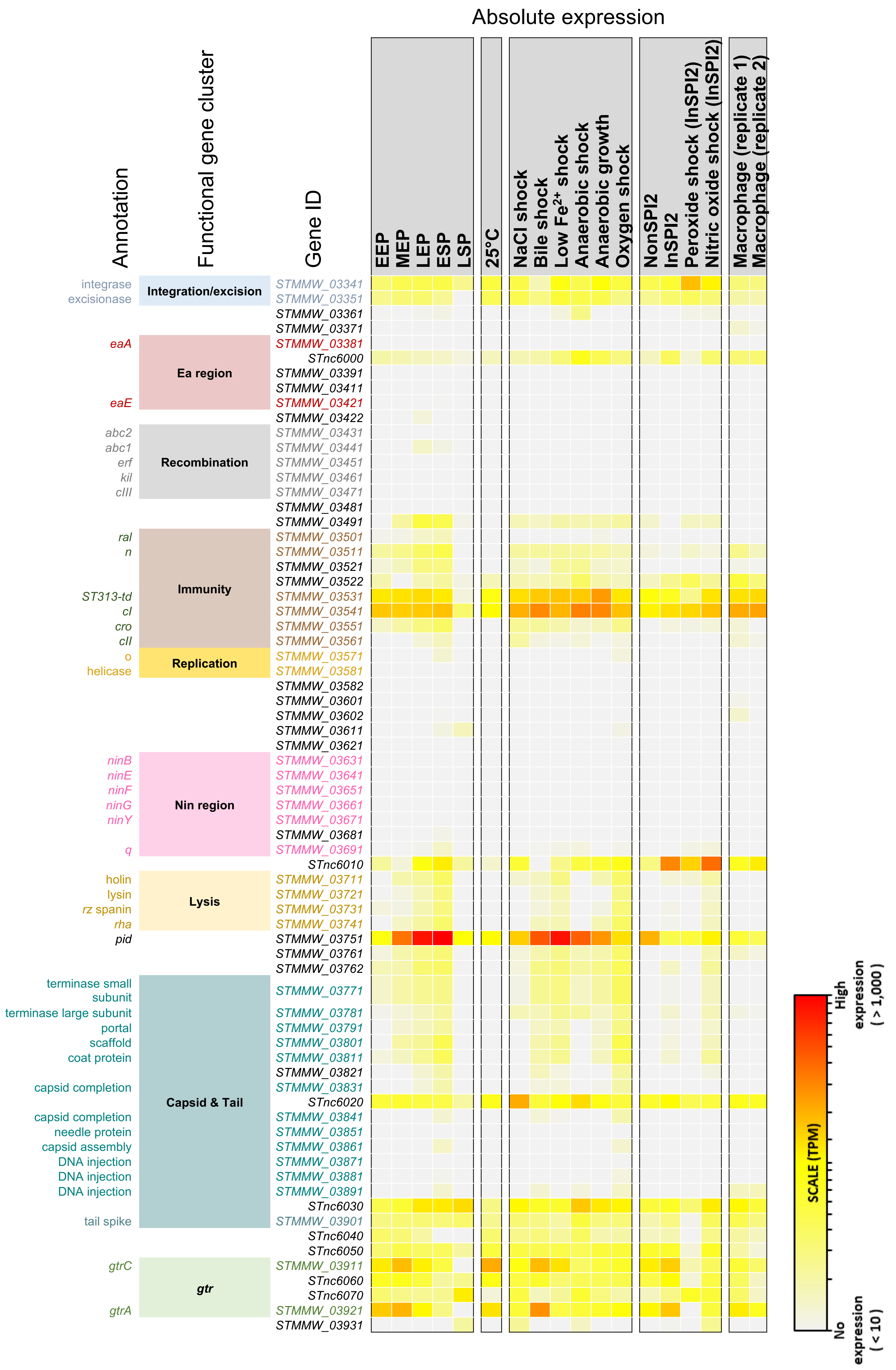

Supplementary Figure 1. Heat map showing the absolute expression of BTP1 genes in 17 infection-relevant conditions. RNA-seq data from Canals et al., 2019 were used to generate absolute expression values (transcript per million, TPM) for each coding gene and annotated ncRNA of the BTP1 prophage.

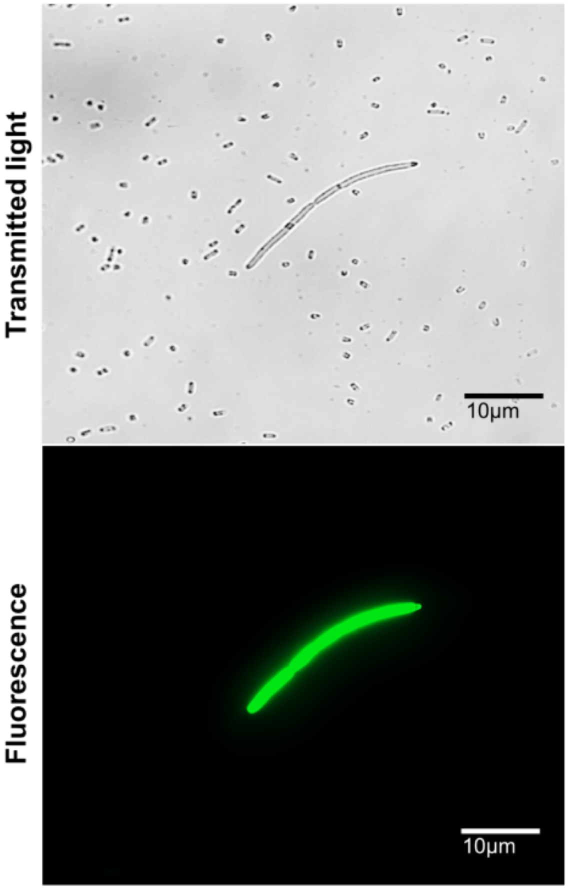

Supplementary Figure 2. GFP reporting of BTP1 prophage induction reveals spontaneous induction occurs in a fraction of the lysogenic population. Example of elongated fluorescent cell morphology found in overnight culture of D23580 Δlysis::*gfp*$^+$. Both transmitted light and fluorescent light images of the same field using a 100X oil immersion objective are shown.

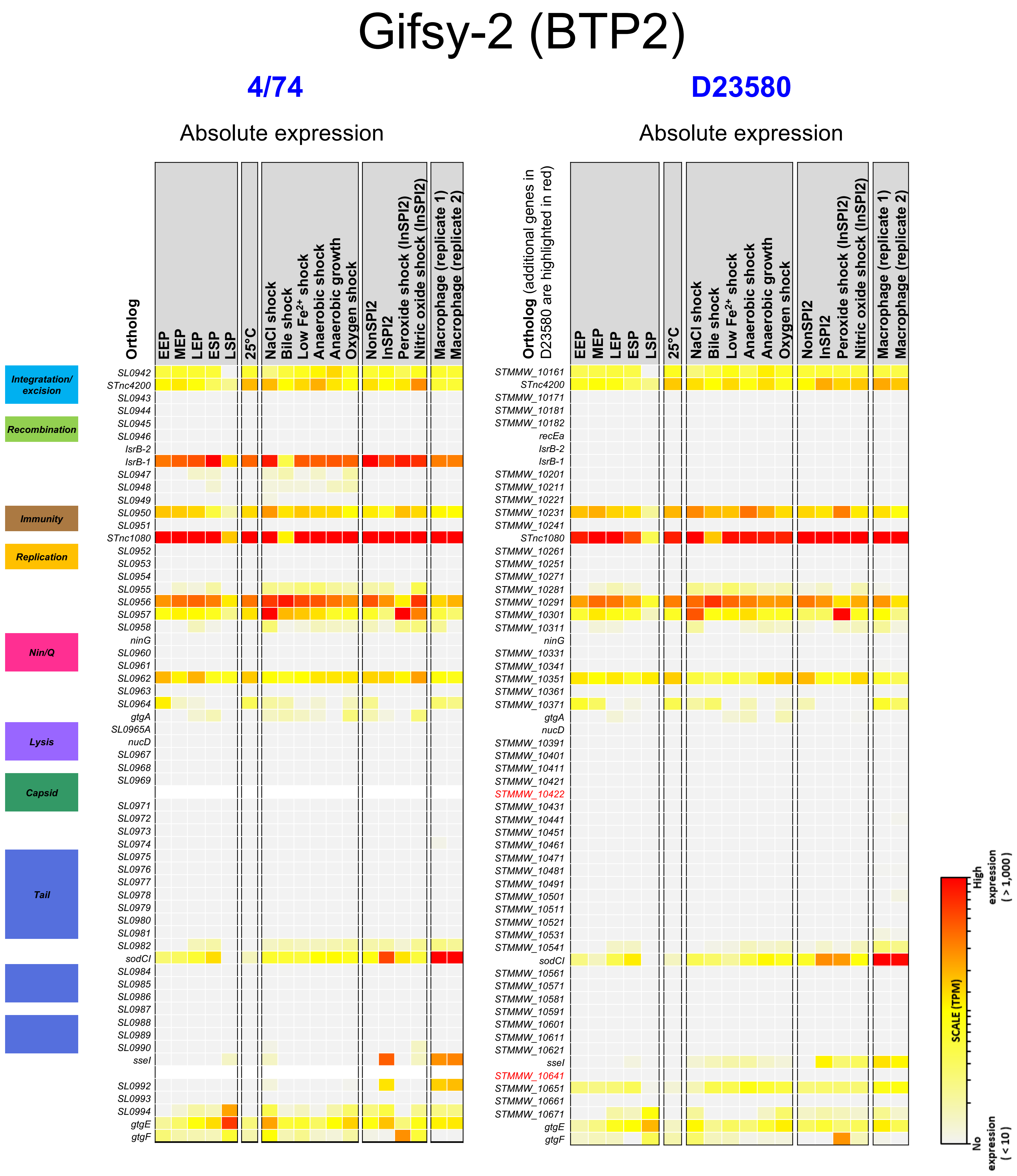

Supplementary Figure 3. Heat map showing the absolute expression of the Gifsy-2 prophage of D23580 and 4/74 strains in 17 infection-relevant conditions. RNA-seq data from Canals et al., 2019 were used to generate absolute expression values (transcript per million, TPM) for each coding gene and annotated ncRNA of the Gifsy-2 prophage. Ortholog IDs in red text indicate genes unique to Gifsy-2^{D23580} or Gifsy-2$^{4/74}$.

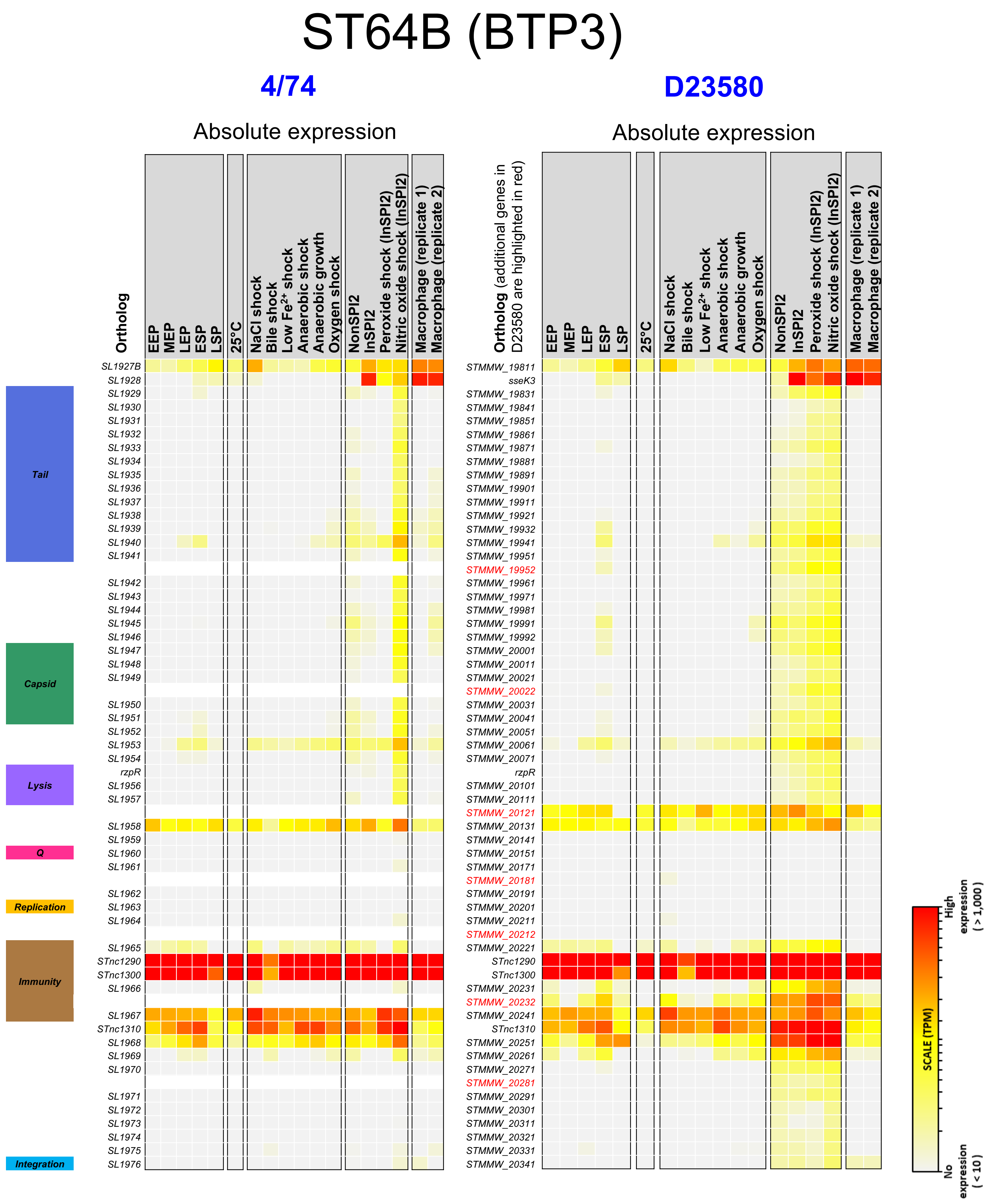

Supplementary Figure 4. Heat map showing the absolute expression of the ST64B prophage of D23580 and 4/74 strains in 17 infection-relevant conditions. RNA-seq data from Canals et al., 2019 were used to generate absolute expression values (transcript per million, TPM) for each coding gene and annotated ncRNA of the ST64B prophage. Ortholog IDs in red text indicate genes unique to ST64B^{D23580} or ST64B$^{4/74}$.

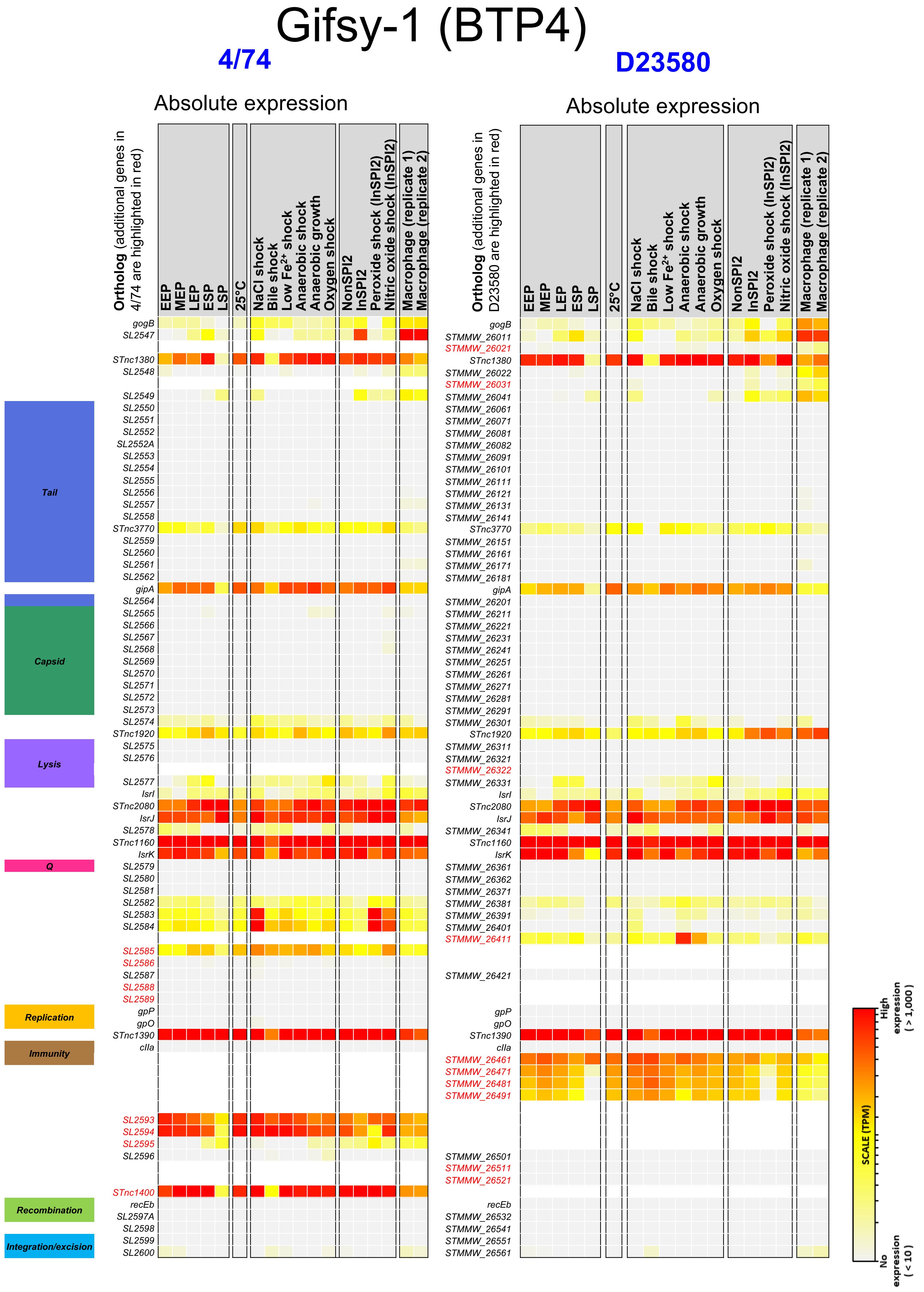

Supplementary Figure 5. Heat map showing the absolute expression of the Gifsy-1 prophage of D23580 and 4/74 strains in 17 infection-relevant conditions. RNA-seq data from Canals et al., 2019 were used to generate absolute expression values (transcript per million, TPM) for each coding gene and annotated ncRNA of the Gifsy-1 prophage. Ortholog IDs in red text indicate genes unique to Gifsy-1^{D23580} or Gifsy-1$^{4/74}$.

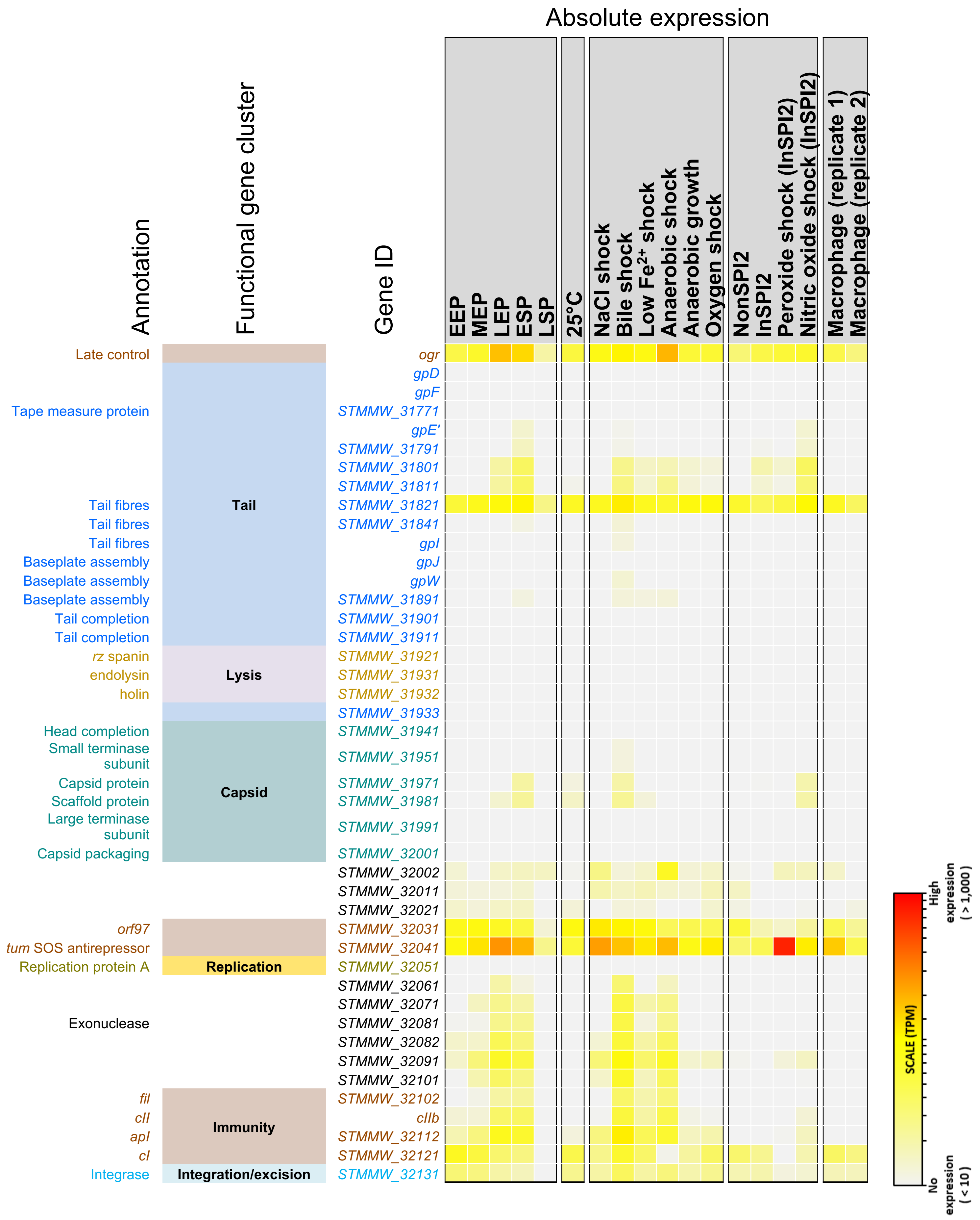

Supplementary Figure 6. Heat map showing the absolute expression of BTP5 genes in 17 infection-relevant conditions. RNA-seq data from Canals et al., 2019 were used to generate absolute expression values (transcript per million, TPM) for each coding gene of the BTP5 prophage.

Supplementary Table 1. Accession numbers of all sequence data used in this study

Accession number	Data type	Description
FN424405	Genome	D23580 genome
CP002487	Genome	4/74 genome
GSM3381722	RNA-seq	4/74 EEP
GSM3381737	RNA-seq	4/74 MEP
GSM3381731	RNA-seq	4/74 LEP
GSM3381726	RNA-seq	4/74 ESP
GSM3381733	RNA-seq	4/74 LSP
GSM3381715	RNA-seq	4/74 25°C
GSM3381738	RNA-seq	4/74 NaCl_S
GSM3381721	RNA-seq	4/74 Bile_S
GSM3381732	RNA-seq	4/74 LwFe_S
GSM3381720	RNA-seq	4/74 No_O2_S
GSM3381743	RNA-seq	4/74 No_O2
GSM3381744	RNA-seq	4/74 O2_S
GSM3381742	RNA-seq	4/74 noSPI2
GSM3381730	RNA-seq	4/74 inSPI2
GSM3381745	RNA-seq	4/74 H2O2_S
GSM3381743	RNA-seq	4/74 NOs
GSM3381734	RNA-seq	4/74 MAC-1
GSM3381735	RNA-seq	4/74 MAC -2
GSM3381689	RNA-seq	D23580 EEP
GSM3381704	RNA-seq	D23580 MEP
GSM3381698	RNA-seq	D23580 LEP
GSM3381693	RNA-seq	D23580 ESP
GSM3381700	RNA-seq	D23580 LSP
GSM3381682	RNA-seq	D23580 25°C
GSM3381705	RNA-seq	D23580 NaCl_S
GSM3381688	RNA-seq	D23580 Bile_S
GSM3381699	RNA-seq	D23580 LwFe_S
GSM3381687	RNA-seq	D23580 No_O2_S
GSM3381686	RNA-seq	D23580 No_O2
GSM3381711	RNA-seq	D23580 O2_S
GSM3381709	RNA-seq	D23580 noSPI2
GSM3381697	RNA-seq	D23580 inSPI2
GSM3381714	RNA-seq	D23580 H2O2_S
GSM3381710	RNA-seq	D23580 NOs
GSM3381701	RNA-seq	D23580 MAC-1
GSM3381702	RNA-seq	D23580 MAC-2
GSM2889540	dRNA-seq	D23580 ESP TEX
GSM2889541	dRNA-seq	D23580 inSPI2 TEX
GSM2889542	dRNA-seq	D23580 pool TEX
GSM4204389	dRNA-seq	D23580 MAC-1 TEX
GSM4204390	dRNA-seq	D23580 MAC-2 TEX

Supplementary Table 2. Description of RNA-seq experiment growth conditions as described in Kröger et al., 2013 and Canals et al., 2019.

**RNA-seq experiment**	**Abbreviation**	**Growth conditions**
Early exponential phase	EEP	Growth in Lennox broth to OD_{600} 0.1
Mid exponential phase	MEP	Growth in Lennox broth to OD_{600} 0.3
Late exponential phase	LEP	Growth in Lennox broth to OD_{600} 1.0
Early stationary phase	ESP	Growth in Lennox broth to OD_{600} 2.0
Late stationary phase	LSP	Growth in Lennox broth to OD_{600} 2.0 + 6 h
Low temperature	25°C	Growth in Lennox broth to OD_{600} 0.3 at 25°C (It's growth at low temperature (ON culture 37°C, 200 RPM, Lennox, diluted 1:1000 and grown at 25 °C until OD_{600} = 0.3)
Osmotic shock	NaCl_S	Growth in Lennox broth to OD_{600} 0.3; then addition of NaCl to a final concentration of 0.3 M for 10 min
Addition of bile	Bile_S	Growth in Lennox broth to OD_{600} 0.3; then addition of bile to a final concentration of 3% for 10 min
Iron limitation	LwFe_S	Growth in Lennox broth to OD_{600} 0.3; then addition of 2,2'-dipyridyl to a final concentration of 0.2 mM for 10 min
Anaerobic shock	No_O2_S	Growth in Lennox broth to OD_{600} 0.3 (50 ml), then filled into 50 ml closed centrifuge tube and incubated without agitation for 30 min at 37°C (Falcon tube)
Anaerobic growth	No_O2	Static growth in Lennox broth to OD_{600} 0.3 in a completely filled and closed 50 ml centrifuge tube (Falcon tube)
Aerobic shock	O2_S	Static growth in Lennox broth to OD_{600} 0.3 in a completely filled and closed 50 ml centrifuge tube (Falcon tube); then 15 min aerobic growth (baffled flask, 250 rpm)
SPI2 non-inducing conditions	noSPI2	Growth in PCN medium to OD_{600} 0.3 (pH 7.4, 25 mM Pi)
SPI2 inducing conditions	inSPI2	Growth in PCN medium to OD_{600} 0.3 (pH 5.8, 0.4 mM Pi)
Oxidative stress	H2O2_S	PCN to OD_{600} 0.3, then addition of H_2O_2 to final concentration of 1 mM H_2O_2 for 12 min
Nitric oxide	NOs	Growth in PCN medium to OD_{600} 0.3 (pH 5.8, 0.4 mM Pi); then addition of 250 µM Spermine NONOate for 20 min
RNA from macrophages	MAC	RNA isolated from RAW 264.7 murine macrophages 8 h post infection

Supplementary Table 3. All oligonucleotide sequences used in this study

Oligo Name	Sequence (5'→3')	Purpose
NW_88	CTAAATACATTCAAATATGTATCCGGTCCAACCAGCGGCACCAG	Replacement of *bla* (ap^R) by *aacC1* (Gm^R) in pP_L
NW_89	GTAAACTTGGTCTGACAGTTACCAATTAGGTGGCGGTACTTGGG	Replacement of *bla* (ap^R) by *aacC1* (Gm^R) in pP_L
STnc6030_pPL_F (NW_295)	GTGAGCGGATAACAAGATACTGAGCACAGCAATATAGTCAACCTGAGAAC	For insertion of STnc6030 asRNA into expression plasmid pPL-Gm using overlap extension PCR cloning
STnc6030_pPL_R (NW_296)	GCCTTTCGTTTTATTTGATGCCTCTAGACTGCGTATCTGAAGGGGATTAAG	
STnc6030_T7 (NW_297)	TAATACGACTCACTATAGGGCTGCGTATCTGAAGGGGATTAAG	For synthesis of anti-STnc6030 riboprobe. Use with STnc60_pPLGM_f
STnc6030_pPL_R NW_296	GCCTTTCGTTTTATTTGATGCCTCTAGACTGCGTATCTGAAGGGGATTAAG	Amplification of STnc6030 region from BTP1 prophage
NW_298	TAATACGACTCACTATAGGGCATAGTCAGGAAGAAGTGAT	
STnc6030_pPL_F NW_295	GTGAGCGGATAACAAGATACTGAGCACAGCAATATAGTCAACCTGAGAAC	Used with NW_296 for nested amplificiation of STnc6030 region from amplification product of NW_298 and NW_295
Late_gfp_2_L_f	TTCCTAATTCAATAGAGCAAATCCCCTCAATAAAGGGGGTAGAGCGTTCTAGATTTAAGAAGGAG	
gfp_kan_2_L_r	CTAAGGAGGATATTCATATGGCGACCGGCGCTCAGCTGGA	Amplification of *gfp*+ gene from pZEP08 with flanking ends for replacement of BTP1 lysis genes with *gfp-KmR* by Lambda Red recombineering
Gfp_kan_2_R_f	TCCAGCTGAGCGCCGGTCGCCATATGAATATCCTCCTTAG	Amplification of *frt-aph*-frt from pKD4 with flanking ends for replacement of BTP1 lysis genes with *gfp-KmR* by Lambda Red recombineering
Late_gfp_2_R_r	TAAGATAAATTTACATGGGTGCTTGTCACCCATGTTTTACAATATGTGTAGGCTGGAGCTGCTTC	
Late_gfp_ext_f	AACACAGTATCCTGGATTTGTTCTA	Amplification of region external to lysis genes to confirm insertion of the gfp-KmR cassette
Late_gfp_ext_r	TGCTTAACAAGGGTAGGTGATGGCC	

Supplementary Table 4. All bacterial strains, phages and plasmids used in this study

**Type**	**Name**	**Description**	**Reference / origin / accession (if available)**
Bacterial strain	JH3621	*S.* Typhimurium D23580 WT	(Kingsley et al., 2009) / FN424405
Bacterial strain	JH3810	*S.* Typhimurium D23580 ΔBTP1	(Owen et al., 2017)
Bacterial strain	JH3676	*S.* Typhimurium 4/74 WT	(Rankin and Taylor, 1966) / CP002487
Bacterial strain	*E. coli* TOP10	Highly competent *E. coli* strain for cloning procedures	Invitrogen
Phage	BTP1 WT		Isolated from D23580 supernatant / LT714109.2
	P22 WT		Gift from A. Aertsen / BK000582
Plasmid	pP_L (pJV300)	Expression plasmid, Ap^R	(Sittka et al., 2007)
Plasmid	pME4510	Promoter search vector pME4510, Gm^R	(Rist et al., 1998)
Plasmid	pP_L-STnc6030	Expression plasmid; STnc6030 sRNA under the control of the $P_{LlacO-1}$ promoter; Gm^R	This study
Plasmid	pP_L-Gm	Derived from pJV-300 but *bla* gene replaced with *aacC1* gene from pME4510; Gm^R	This study
Plasmid	pZEP08	Used as template to amplify the *gfp*+ for construction of a prophage induction reporter construct	(Hautefort et al., 2003)
Plasmid	pKD4	*aph-cassette* template plasmid ; Km_R	(Datsenko and Wanner, 2000)
Plasmid	pSIM-5-tet	Lambda Red recombineering plasmid	(Koskiniemi et al., 2011)
